# The Mutational Landscape of Acute Promyelocytic Leukemia Reveals an Interacting Network of Co-Occurrences and Recurrent Mutations

**DOI:** 10.1371/journal.pone.0148346

**Published:** 2016-02-17

**Authors:** Mariam Ibáñez, José Carbonell-Caballero, Luz García-Alonso, Esperanza Such, Jorge Jiménez-Almazán, Enrique Vidal, Eva Barragán, María López-Pavía, Marta LLop, Iván Martín, Inés Gómez-Seguí, Pau Montesinos, Miguel A. Sanz, Joaquín Dopazo, José Cervera

**Affiliations:** 1 Hematology Service, Hospital Universitario y Politécnico La Fe, Valencia, Spain; 2 Computational Genomics Department, Centro de Investigación Príncipe Felipe, Valencia, Spain; 3 Laboratory of Molecular Biology, Department of Clinical Chemistry, Hospital Universitario La Fe, Valencia, Spain; 4 Functional Genomics Node, Spanish National Institute of Bioinformatics at CIPF, 46012, Valencia, Spain; 5 Bioinformatics of Rare Diseases (BIER), CIBER de Enfermedades Raras (CIBERER), Valencia, Spain; 6 Genetics Unit, Hospital Universitario y Politécnico La Fe, Valencia, Spain; German Cancer Research Center, GERMANY

## Abstract

Preliminary Acute Promyelocytic Leukemia (APL) whole exome sequencing (WES) studies have identified a huge number of somatic mutations affecting more than a hundred different genes mainly in a non-recurrent manner, suggesting that APL is a heterogeneous disease with secondary relevant changes not yet defined. To extend our knowledge of subtle genetic alterations involved in APL that might cooperate with *PML/RARA* in the leukemogenic process, we performed a comprehensive analysis of somatic mutations in APL combining WES with sequencing of a custom panel of targeted genes by next-generation sequencing. To select a reduced subset of high confidence candidate driver genes, further *in silico* analysis were carried out. After prioritization and network analysis we found recurrent deleterious mutations in 8 individual genes (*STAG2*, *U2AF1*, *SMC1A*, *USP9X*, *IKZF1*, *LYN*, *MYCBP2* and *PTPN11*) with a strong potential of being involved in APL pathogenesis. Our network analysis of multiple mutations provides a reliable approach to prioritize genes for additional analysis, improving our knowledge of the leukemogenesis interactome. Additionally, we have defined a functional module in the interactome of APL. The hypothesis is that the number, or the specific combinations, of mutations harbored in each patient might not be as important as the disturbance caused in biological key functions, triggered by several not necessarily recurrent mutations.

## Introduction

Acute Promyelocytic Leukemia (APL) is characterized by the *PML/RARA* rearrangement as a consequence of the translocation t(15;17)(q24;q21). However, it is well established that this aberration alone is not able to trigger the whole leukemic phenotype[1]. Additional chromosomal abnormalities to t(15;17) and other gene mutations (e.g. *FLT3* mutations) have been reported to act as potential secondary hits in up to 40% of APL cases. [2] However, little is known about the nature and precise role of these cooperating events in APL [3].

The recent advent of next-generation sequencing (NGS) technologies has allowed for the identification of a growing number of novel mutations in leukemia and other cancers. In APL, preliminary whole genome/exome studies involving about thirty APL patients identified several somatic mutations affecting up to 135 different genes in a non-recurrent manner, except for *FLT3*, *WT1* and *KRAS*. These findings suggest that APL is a heterogeneous disease with secondary relevant changes not yet defined [4–8]. Recent approaches that take into account the physical and functional interactions of the mutated genes have been useful to discover driver mutations in several cancers [8–11].

To extend our knowledge of subtle genetic alterations involved in APL that might cooperate with *PML/RARA* in the leukemogenic process, we have performed whole-exome sequencing (WES) of diagnosis-completed remission matched samples on a selected discovery cohort of *de novo* APL patients and targeted resequenced novel candidate genes in an extended cohort. Finally, we used network analysis to select a reduced subset of high confidence candidate driver genes.

## Material and Methods Summary

Whole-exome sequencing (WES) of diagnosis-completed remission matched samples from 5 APL patients (discovery cohort) was performed to identify gene mutations. WES data were analyzed using an in-house bioinformatics pipeline (Data in S1 Material and Methods and S1 Fig) to compare the coding sequence of matched samples and identify somatically acquired deleterious changes. The variants detected were confirmed in both samples of each patient by Sanger sequencing. Furthermore, complete coding sequence of 97 genes (17 novel candidate genes with confirmed somatic mutations from in-house results and 80 genes reported to be mutated in at least 1 patient from previous APL studies [4, 6]) were targeted re-sequenced in 25 additional patients (extended cohort) using SureDesign Tool (Agilent) for NGS, according to the manufacturer’s instructions. Finally, network enrichment analysis [12, 13] was used to help in the prioritization of candidate genes on the basis of their connectivity, mutational recurrence, mutational co-occurrence and cancer related functionalities.

According to the Declaration of Helsinki, written informed consent was obtained from all patients, and the protocol was approved by the Research Ethics Board of the Health Research Institute Hospital La Fe (No.2012/0175).

Further Material and Methods details are provided in the Extended Experimental Procedures at the S1 Material and Methods.

## Results

### Whole Exome Sequencing

Average target coverage obtained in both diagnostic and remission samples was 61.5x (S1 Table). In addition, 81% of the target regions were covered by more than 10 reads, with a high concordance (~98%) for detected SNPs from array assays (Data in S2 Fig). A total of 309,498 variant positions were detected in all sequenced exons from all the samples (Table 1).

**Table 1:** SNV and indel filtering steps for identification of somatic variants.

Filter	APL_1	APL_2	APL_3	APL_4	APL_5	Total
Variants detected	55320	69713	64395	58459	61611	309498
Somatic	4809	5824	5956	5015	6288	27892
Absent in CR	3814	5026	4954	4340	5160	23294
High quality	29	19	18	9	21	96
Coding (SNVs+indels)	20 (14+6)	11 (10+1)	12 (9+3)	8 (4+4)	13 (9+4)	64 (46+18)
Deleterious (SNVs+indels)	14 (8+6)	7 (6+1)	10 (7+3)	8 (4+4)	11 (7+4)	50 (32+18)
Unknown in dbSNP	14	5	8	7	10	44

CR. Complete remission

After a strict filtering process (Data in S1 and S3 Figs), a total of 50 high quality candidate somatic variants (32 non-synonymous coding SNVs and 18 small indels) were found (Table 1). The distribution of the number of variants per exome showed an average of 10 mutations per sample (range 7–14). Recurrent mutations or common mutated genes were not observed.

### Validation of the candidate variants in the discovery cohort

Sanger sequencing of samples from the discovery cohort confirmed 18 of the variant candidates (17 missense coding mutations and 1 indel) in 17 genes. The affected genes were *ADC*, *ALPK3*, *APPL1*, *CSNK1A1L*, *FAM171A1*, *FBLN1*, *FILIP1L*, *FLT3* (carrying 2 mutations), *GJB7*, *HMGCR*, *KIAA0317*, *MDN1*, *NR4A2*, *ORC3*, *PRICKL2*, *PTPRT* and *ZNF518B* (S2 Table). No recurrent mutations were observed except for *FLT3* mutations, neither when the mutations were checked in an independent cohort of 65 *de novo* APL patients. The remaining variations have not been reported before in APL cases. The mutational spectrum was dominated by C>T/G>A transitions.

Among the 17 validated mutated genes containing at least one mutation, one gene was involved in the splicing machinery (*ADC*), 5 in cell cycle (*ORC3*, *PRICKLE2*, *PTPRT*, *ZNF518B*, *APPL1*), 2 in protein modifications (*ALPK3*, *CSNK1AL*), 1 protein kinase (*FLT3*) and 8 in other cell functions (*FBLN1*, *FAM171A1*, *FILIP1L*, *GJB7*, *HMGCR*, *KIAA0317*, *MDN1*, *NR4A2*).

### Screening for somatic mutations in the complete coding sequence of 97 genes in the extended cohort

Details of the 25 patients included in the extended cohort are provided in S3 Table and the genes included in the targeted resequencing are described in S4 Table. Overall, mean target coverage was 223.5x. Absolute median coverage at positions where mutations were identified was 208.4x.

**Fig 1:**
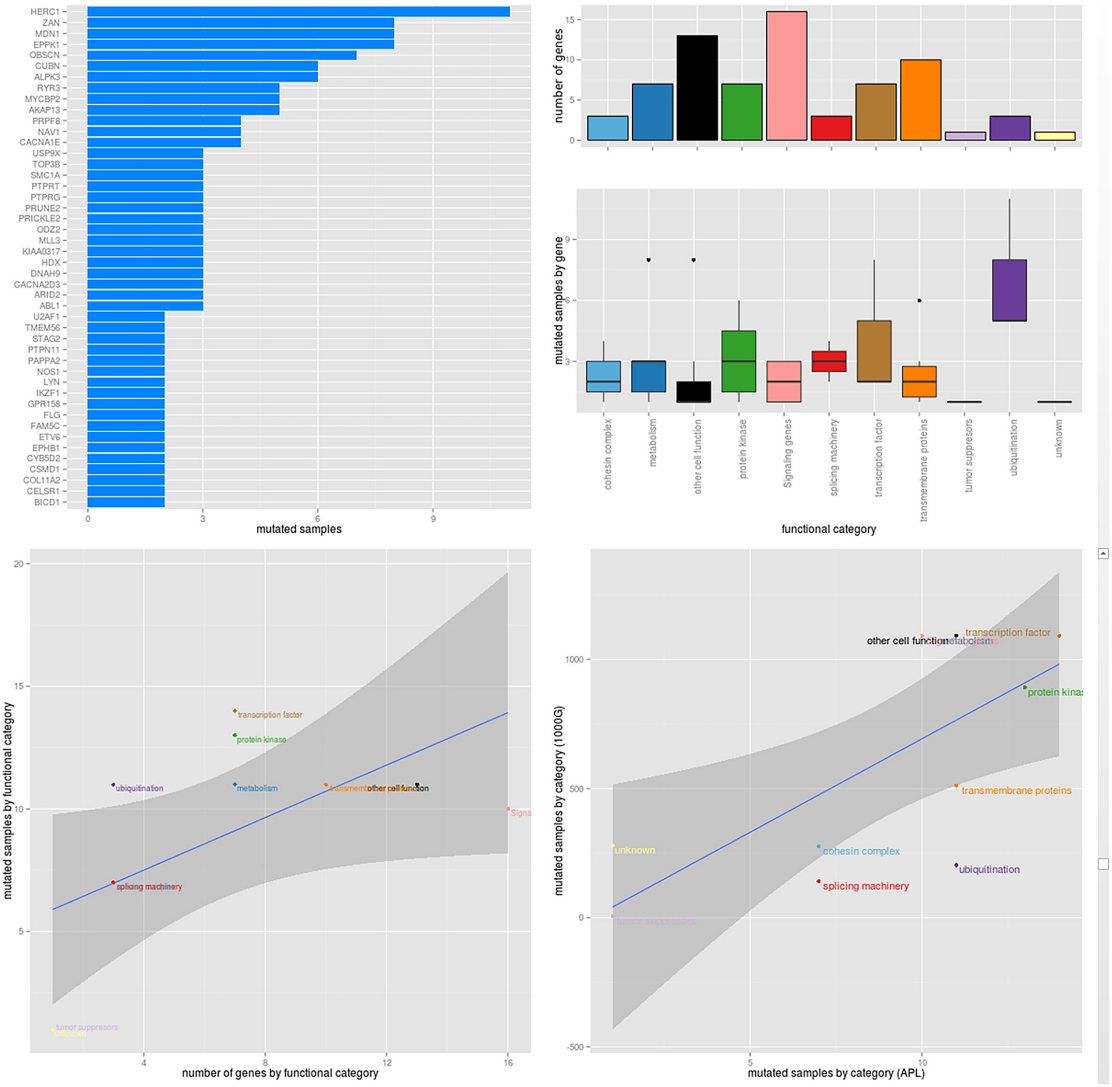
Distribution of selected mutations along the different affected genes and their related functional categories. A) Number of mutated samples by gene according to the described mutation filtering protocol (S2 Fig). Only 33 recurrent genes were included. B) Number of mutated samples by gene category (bottom). The corresponding number of genes included in each functional category (top) is also represented in order to avoid any size bias. C) The functional categories are distributed depending on the observed mutated samples and the known number of belonging genes. Both transcription factor and ubiquitination categories shown and excess of mutated samples. D) The number of mutated samples per category in the haloplex cohort were compared against a reference population of healthy samples (1000 genomes). Here, while metabolism and signalling genes categories appear poorly mutated, ubiquitination appears remarkably more mutated than the reference population.

After the filtering procedure (Data in S3 Fig) we identified a total of 219 high-confidence variants comprising nonsynonymous variants (n = 192; 88%), reading frameshifts (n = 3; 1%), stop gain codons (n = 14; 6%), variants affecting the splicing (9; 4%) and also affecting to ncRNAs (1; 0.5%) (S5 Table). A mean of 9.48 mutations per sample (range 1–47) were detected. The ratio transition/transversion was 0.63. The variants affected 72 different genes. Of these, 46 were mutated in more than one patient (range 2–15) and 39 genes carried the same mutation in several samples (Fig 1A). *HERC1* was the most recurrently mutated (44%), followed by *ZAN* (32%), *MDN1* (32%), *EPKK1* (32%) and *OBSCN* (28%). We detected 15 mutated genes [*HERC1* (44%), *CACNA2D3* (20%), *MYCBP2* (20%), *CACNA1E* (16%), *TOP3B* (12%), *SMC1A* (12%), *USP9X* (12%), *KIAA0317* (12%), *PRICKLE2* (8%), *IKZF1* (8%), *LYN* (8%), *TMEM56* (8%), *U2AF1* (8%), *PTPN11* (8%) and *STAG2* (8%)] at a higher frequency in our cohort than expected in the 1000 genomes repository [14] (*P* ≤ 0.05) (Table 2). Among them, 2 genes, *PRICKLE2* and *KIAA0317*, were detected in the WES results of our discovery cohort, and in both cases, the mutations were spread out along the gene (S5 Table).

**Table 2:** Mutated genes with a higher frequency in our cohort than expected in the 1000 genomes repository.

Gene	APL frequency (%)	1000g frequency (%)	Functional Category	Description	Expression in Bone Marrow
*HERC1*	44	3	ubiquitination	Stimulates guanine nucleotide exchange on ARF1 and Rab proteins. This protein may be involved in membrane transport processes	medium
*CACNA2D3*	12	0.9	transmembrane protein	Acts as a regulatory subunit for calcium channel	medium
*CACNA1E*	16	0.6	protein kinase	Mediates the entry of calcium ions into excitable cells and is also involved in a variety of calcium-dependent processes, including muscle contraction, hormone or neurotransmitter release, gene expression, cell motility, cell division and cell death	none
*MYCBP2*	20	2	ubiquitinitation	Involved in the ubiquitination and subsequent proteasomal degradation of target proteins. May function as a facilitator or regulator of transcriptional activation by MYC.	high
*KIAA0317*	12	0.4	ubiquitinitation	E3 ubiquitin-protein ligase which inhibits apoptosis by ubiquitinating and targeting for degradation.	medium
*SMC1A*	12	0	cohesion complex	Central component of cohesion complex that is required during cell cycle and in DNA repair.	high
*USP9X*	12	0.09	splicing	Involved in the processing of ubiquitin precursors and proteins, that may play an important regulatory role preventing degradation of proteins.	high
*TOP3B*	12	0.9	transcriptional factor	Releases the supercoiling and torsional tension of DNA.	medium
*PRICKLE2*	8	0.1	other cell functions	The function is not known, however, may be involved in seizure prevention	medium
*IKZF1*	8	0	transcription regulator	As a transcription regulator of hematopoietic cell differentiation, plays a role in the development of lymphocytes, B- and T-cells.	high
*LYN*	8	0	tyrosine-protein kinase	Negative regulator that plays an important role in the regulation of B-cell differentiation, proliferation, survival and apoptosis, and is important for immune self-tolerance. Acts as an effector of EPOR (erythropoietin receptor) in controlling KIT expression and may play a role in erythroid differentiation during the switch between proliferation and maturation.	high
*STAG2*	8	0.01	cohesin complex	Component of cohesin complex, a complex required for the cohesion of sister chromatids after DNA replication	high
*PTPN11*	8	0.01	transmembrane protein	Acts downstream of various receptor and cytoplasmic protein tyrosine kinases to participate in the signal transduction from the cell surface to the nucleus.	high
*U2AF1*	8	0	splicing	Plays a critical role in both constitutive and enhancer-dependent splicing by mediating protein-protein interactions and protein-RNA interactions required for accurate 3'-splice site selection	high
*TMEM56*	8	0	transcriptional factor	Transmembrane protein who acts as a transcriptional factor	low

### Functional categorization of mutated genes

The 46 recurrently mutated genes were also analyzed for functional enrichment of gene ontology terms [15] and grouped according to their biological function (Fig 1B). We observed an interaction pattern between different functional categories. Patients harboring mutations in a gene of the ubiquitination functional category also had mutations in metabolism (n = 8) or signaling (n = 8) or spliceosome (n = 6x). Furthermore, patients with mutations in transmembrane protein genes carried mutations in signaling genes (n = 7) or in spliceosome (n = 6) or in protein kinase (n = 10). Additionally, we found patients with mutations in signaling genes and spliceosome (n = 6) or in the metabolism and signaling genes (n = 8). All the described interactions were statically significance (*P* < 0.05).

In addition, the protein kinase, ubiquitination and the transcription factor category were more frequent than those awaited by the number of genes that compiled each category (Fig 1B, 1C and 1D). The same observation was made for the ubiquitination and spliceosome category, when we compared our results with those expected in the 1000 genomes repository [14].

#### Candidate gene prioritization

**Fig 2:**
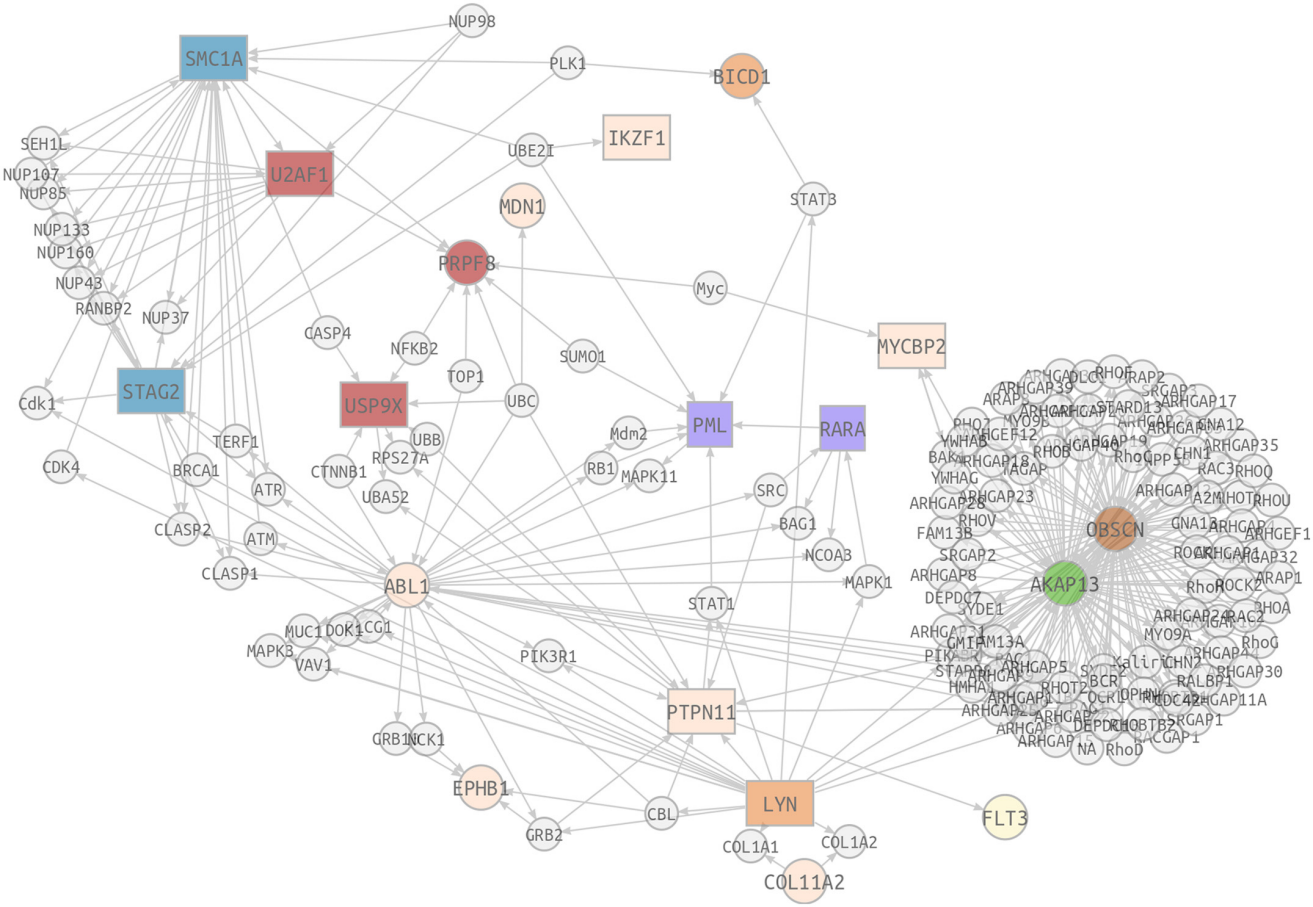
Network-based analysis (SNOW) applied to 46 selected genes (33 recurrent and *RARA*, *PML*, and *FLT3* genes). The network was complemented with the co-occurrence relationships, in order to summarize the two kind of significant results. Significant network-based analysis genes are coloured in light blue and stroked with a magenta border whether they resulted also significant in co-occurrence analysis. Genes only included by co-occurrence are coloured in magenta. Intermediate genes were painted in white and square shaped. While grey edges represent protein-protein interaction, relationships, broad orange dashed lines describe significant co-occurrences. Moreover, main genes are grouped depending on their biological role (cohesin complex, signalling pathways, spliceosome, RHO-GTPase, retinoic acid regulators and other cellular processes roles).

Network analysis [12, 13], as implemented in the Babelomics [16] tool, was also used to prioritize the best candidate genes. For this propose, we included the set of recurrently mutated genes from NGS results beside *PML*, *RARA* and *FLT3*-ITD data of each patient obtained from cytogenetic and molecular analysis performed at diagnosis time. So, 17 out of the 46 selected genes, together with *PML* and *RARA*, were found to be significantly more connected that the random expectation (*P =* 0.02) (Fig 2). Some of the genes (*STAG2*, *U2AF1*, *SMC1A*, *USP9X*, *IKZF1*, *LYN*, *MYCBP2* and *PTPN11*) also displayed an accumulation of mutations significantly higher that the corresponding healthy controls taken from the 1000 genomes repository [14]. Among these genes, the functional implications of the network could be observed in the edges connecting well-known cancer-related genes with similar functional annotations. *PTPN11* interacted with *PML* through an intermediate gene, *STAT1*. At the same time, another member of the STAT family, *STAT3*, networked with *PML* and with a gene that works as a transmembrane protein (*LYN*). Moreover, we have identified an edge relating the genes with transmembrane protein function with those from the spliceosome (*U2AF1*, and *USP9X*) and the cohesin complex (*SMC1A* and *STAG2)* that have an important role in cancer. Concurrently, the cohesin complex was joined by the nuclear pore complex proteins (such as *NUP98)* with the spliceosome, which networked with *PML* through a transcription factor binding and with the Rho GTPase cycle through signaling pathways (*MYCBP2*).

We reassessed the value of our network analyses by adding the mutations reported in APL cases of TCGA dataset. The combination of both cohorts (n = 45) displayed an accumulation of mutations in some genes. Several genes without recurrence in both series were enriched for mutations so we finally obtained an input of 59 genes. Despite the increase in the number of genes, the 8 candidate genes (*STAG2*, *U2AF1*, *SMC1A*, *USP9X*, *IKZF1*, *LYN*, *MYCBP2* and *PTPN11*) obtained from our cohort analysis remained significantly involved in the network (Data in S4 Fig).

### Co-occurrence of SNVs

**Fig 3:**
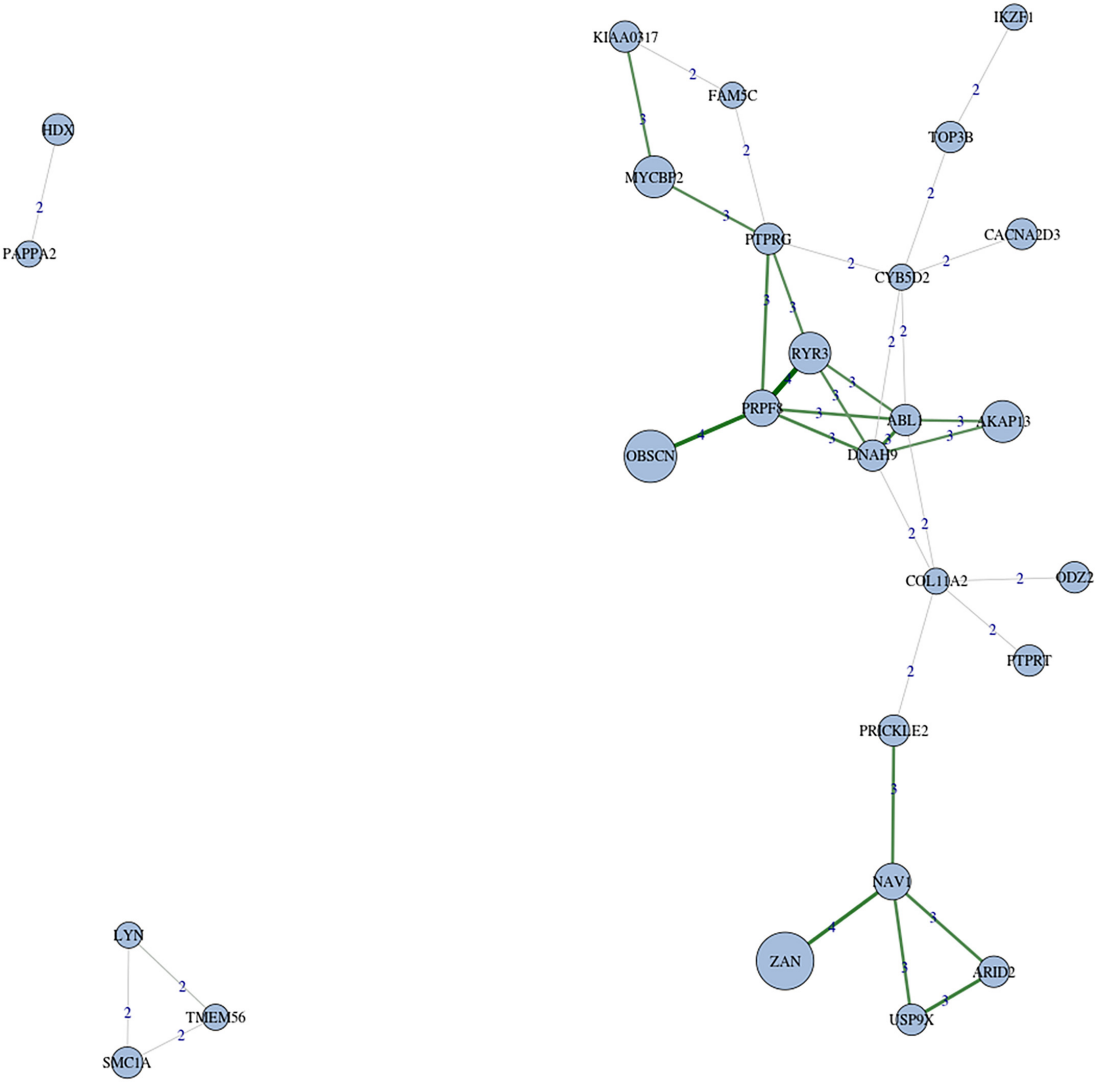
Network of significant gene co-occurrences. Genes are represented by nodes and their sizes defined from the number of significant co-occurrences they are implied. Edges represent co-occurrences between pairs of genes. Every edge is labelled with the number of samples that carries the mutated pair of genes as follows: higher than expected co-occurrences are coloured in green, while lower than expected (only one) in red. Edge width is proportional to the statistical p-value of chi-square test. Those genes co-occurring only at one single patient are painted in white. Seven co-occurrence subnetworks arise from the significant co-occurrence network, where remarkably a single component connected the half of represented genes. In contrast, 3 pairs are simultaneously mutated only in 2 different individuals, and 3 significant co-occurrence subnetworks, only in 1 patient.

To assess patterns of co-occurrence we focused on the 46 genes mutated in more than one patient. Fig 3 depicts the presence of concomitant mutations within single patients, where multiple pairs of genes showed co-occurring mutations. We discovered a total of 32 interactions of mutations occurring at the same time in 2 or more patients with statistical significance (*P* ≤ 0.05). Interestingly, many of these co-occurrences took place within the significant subnetwork of protein interactions that link many of the affected genes. For example, *PRPF8* mutations were carried simultaneously with mutations in *ABL*, *DNAH9* and *PTPRG*. Mutations in *PTPRG* coincided with mutations in other two genes of the network, *MYCBP2* and *KIAA0317*. Moreover mutations were simultaneous in *SMC1A* and *LYN2* (Fig 3). This association was not replicated in the 1000 genomes repository [14].

### Correlation with clinical data

No single mutation or functional category showed an association with clinical variables or prognostic impact in terms of OS or RFS.

## Discussion

This study shows a comprehensive analysis of APL, combining WES with the assessment of somatic mutations by a custom next-generation sequencing panel of targeted genes. It represents, to our knowledge, the largest analysis of somatic alterations in this subtype of acute leukemia. After careful filtering of variants, we identified deleterious mutations in eight genes previously reported as mutated in other myeloid neoplasms. Network enrichment analysis significantly points to these 8 recurrently mutated genes to be involved in the pathogenesis of APL.

To identify leukemia-specific somatic alterations that could cooperate in the development and progression of leukemia [6, 7], WES analysis was performed in 5 of diagnosis-completed remission matched samples. We examined DNA from bone marrow or peripheral blood at complete remission of each single case (defined according to the recommendations of Cheson *et al*.). Using a matched remission sample as a germline control we could established the *de novo* nature of each anomaly and eliminate false positive results that correspond to low-frequency variations that may not be reported in public SNPs databases. Matched samples were systematically covered by more than 10 reads, previously reported to be sufficient for variant calling [17]. In parallel, to minimize errors associated with any measurement, diploid coverage for matched samples was assessed using a set of high-quality SNPs derived from an array platform. We detected a 98% of concordance among the 1% of the SNPs common for the WES and the SNP-A approach. The small differences observed could be due to the fact that SNP-A focuses exclusively on SNP´s position, while WES screens the whole exome with different coverage in different regions. WES regions with low coverage are filtered by the designed pipeline, missing SNPs therein. In addition to the stringent filtering criteria, to prevent false-positive calls in WES, all alterations were analyzed by direct sequencing in paired diagnosis and complete remission samples. Only a part of the initially detected variants were confirmed, probably due to the limited sensitivity of Sanger sequencing, able to detect mutations that are present in at least 20% of the analyzed cells, or to the introduction of some artefacts during the WES amplification. Moreover, some false negative results were possible, due to the difficulty in the identification of indels or the presence of low frequency alleles. As a whole, these technical limitations might imply an underestimation of candidate mutated genes in our discovery cohort.

In a first step, we identified 18 genetic variants none of them, with the exception of *FLT3*, previously known to be mutated in leukemia. When we extended the validation of these candidate variants to 65 patients from an independent cohort, none of the newly discovered mutations were found to be recurrent. The same was found when we compared our mutated genes with those previously reported by the TCGA study and from APL patients previously described [4, 7]. This is in agreement with recent reports, where no recurrent mutations were found [4, 6, 7]. It is also consistent with current models of leukemogenesis in APL, which hold that the vast majority of mutations occur as random events in normal precursor cells before these cells acquire an initiating mutation, so only a small portion of the alterations detected in each APL exome might be cooperating events, acting *PML-RARA* as the main driver mutation [6]. Furthermore, recent studies have highlighted that certain cancer types display fewer mutations on average. Actually, it has been shown that in leukemia the number of somatic mutations harbored by patient is 3 to 7 times lower that in solid tumors [18]. In particular, the study developed by the TCGA consortium brought out that in APL the expected recurrent somatic mutations by patient were almost twice lower than in other subtypes of AML.[4, 6, 7].

To test the relationship between our WES candidate genes and mutations in oncogenes known to be involved in myeloid malignancies, we selected a panel of 97 target genes known to be recurrently mutated in APL with a frequency ≥ 2% [7]. The selected subset of genes was analyzed in an extended cohort of 25 patients using in-house scripts. As no germline matched sample was available from each patient, and to avoid the selection of germline variants, we implemented a germinality test that was also complemented with a comparison against the healthy 1000G cohort and other variant population databases, in order to filter out the majority of germline variants. On the other hand, our protocol takes into account several sequencing artifacts as strand bias and other measurable effects derived from poorly mapped reads, what ensures to minimize the proportion of false positive mutations. The resequencing produced a high level of coverage for the targeted sequences, thus increasing our ability to detect mutations with less than 10% presence. We found a mean of 9.48 mutations (range 1–47) in each patient across 54 genes.

In line with recent reports, highlighting the complex nature of genomic aberrations in leukemia [4, 6, 7], we observed a wide spectrum of different mutations affecting well-known as well as novel APL targets. Of note, we found 8 genes mutated at a frequency significantly higher (8–40%; P<0.01) than expected in the 1000 genomes repository [14], and therefore with a strong novel potential to be involved in APL pathogenesis. With the exception of 4 genes well characterized in hematological neoplasms, we identified several mutations affecting genes without any known evidence to be involved directly in APL. Among them, 2 genes, *PRICKLE2 and KIAA0317*, were also detected in the discovery cohort by WES. Focusing on the allele frequency of each variation, it was not possible to distinguish between those mutations that are likely to be initiating or to be cooperating events. Functional validation studies will be required to assess the importance of these newly defined somatic mutations for APL pathogenesis.

To provide a reliable approach to prioritize genes for additional analysis, we used exploratory *in silico* approaches. First, we grouped mutations into larger sets, or pathways, according to the functional GO categories and examined patterns of mutual co-occurrence or exclusion between these groups. The co-occurring mutations affected mainly genes which were participating in the metabolism, along with genes acting as a protein kinase or as transmembrane proteins, and also mutated genes related to the signaling, spliceosome and ubiquitination functions. Additionally, network analysis resulted in a significantly connected subnetwork in which 17 of the candidate genes, recurrently harboring deleterious mutations, were immersed. These findings reinforce the hypothesis that a functional module, partially defined by this subnetwork, is associated to APL, and that specific combinations of mutations within this module contribute to the arising and maintenance of APL. This result strongly suggests that all these genes are close in the interactome, as frequently occur with genes of the same disease [19, 20]. This confirms that mutations also co-occur in the interactome neighborhood. Genes connected to the candidate genes were involved in several key functionalities, such as cell cycle, kinases, transcriptional factors, splicesome and cohesin complex. These results are in agreement with those previously reported by the TCGA consortium, where APL patients were affected by several mutations in spliceosome genes and other unusually reported myeloid tyrosine kinases and transcriptional factors [4, 6, 7]. It seems that in the development of APL the impairment of a specific relevant gene is not as essential as the interaction of several mutated genes belonging to different functionally related categories. As a result, the combination of the specific APL chromosomal rearrangement with other mutated genes might trigger the leukemogenesis, displaying the importance of this biologic link. This hypothesis could explain the lack of concordance in the set of genes reported by previous studies, where numerous genes have been described to be involved in a not recurrent manner in APL, but with similar cell functions [4, 6, 7].

A major limitation to our study is the relative small sample size. We have analyzed an initial cohort of 5 patients by WES and 25 patients by a targeted gene-panel using NGS. Previous studies have also been conducted in small cohorts, ranged from 1–20 APL patients [4–7]. Although our series is one of the largest APL cohorts (n = 30) analyzed by NGS, it has been difficult to set up a significant independent association with outcome for any gene mutation or functional category, as neither was in similar preceding reports in APL. Therefore, more solid evidence based on larger series with long-term follow-up are needed to correlate our results with other clinical data.

In summary, this report describes the analysis of the largest number of gene mutations in APL carried out to date. After prioritization and network analysis we found 8 individual genes (*STAG2*, *U2AF1*, *SMC1A*, *USP9X*, *IKZF1*, *LYN*, *MYCBP2* and *PTPN11*) with a strong novel potential of being involved in APL pathogenesis. In fact, on the basis of the study of recurrent mutations in interaction interfaces, *PTPN11* has recently been suggested to be a cancer driver [21]. Also, genes *STAG2* and *U2AF1* were detected as drivers in AML and *SMC1A* in distinct cancers by the IntOGen tool [22]. Additionally, we have defined a functional module in the interactome of APL, hypothesizing that the number or the specific combination of mutations harbored in each patient might not be as important as the perturbation caused in biological key functions, triggered by several not necessarily recurrent mutations [23]. Finally, despite our study reports new APL candidate genes, it is clear that the understanding of the total number and clonal distribution of mutations in this disease is still limited and the future potential of discovery of new disease genes is high.

## Supporting Information

S1 Fig In-house bioinformatics pipeline for next generation sequencing data processing. (TIF)

S2 Fig Overview of the common probes from WES analysis and the SNP-A approach of matched samples of each APL patient. (TIF)

S3 Fig Sequencing workflow and bioinformatics pipeline for identification of somatic mutations. After trimming low quality reads and tails, PCR duplicated sequences were filtered. Selected reads, from DNA samples of APL diagnosis and completed remission cells, were aligned against human genome. A series of filters were applied for somatic mutation detection.
(TIF)

S4 Fig Network of significant gene co-occurrences reported in our cohort and in APL cases of TCGA dataset. Genes are represented by nodes and their sizes defined from the number of significant co-occurrences they are implied. Edges represent co-occurrences between pairs of genes. Every edge is labelled with the number of samples that carries the mutated pair of genes as follows: higher than expected co-occurrences are coloured in green, while lower than expected (only one) in red. Edge width is proportional to the statistical p-value of chi-square test. Those genes co-occurring only at one single patient are painted in white. Seven co-occurrence subnetworks arise from the significant co-occurrence network, where remarkably a single component connected the half of represented genes. In contrast, 3 pairs are simultaneously mutated only in 2 different individuals, and 3 significant co-occurrence subnetworks, only in 1 patient.
(TIF)

S1 Material and Methods Further Material and Methods details are provided in the Extended Experimental Procedures at the Supplemental Information. **Table A.** Main characteristics of the discovery cohort of patients. **Table B.** Primers used to validate mutations detected by WES
(DOC)

S1 Table Sequencing results obtained for the samples sequenced (DOCX)

S2 Table Mutations detected by whole exome sequencing in the study cohort. (DOCX)

S3 Table Main characteristics of the extended cohort of patients. (DOCX)

S4 Table Genes included in the targeted resequencing. (DOCX)

S5 Table The mutated genes detected by target-resequencing. (DOCX)

## References

[1] Zimonjic DB, Pollock JL, Westervelt P, Popescu NC, Ley TJ: Acquired, nonrandom chromosomal abnormalities associated with the development of acute promyelocytic leukemia in transgenic mice. *Proc Natl Acad Sci U S A* 2000, 97:13306–13311. 11087871 10.1073/pnas.97.24.13306 PMC27220

[2] Gomez-Segui I, Cervera J, Such E, Martinez-Cuadron D, Luna I, Ibanez M, et al: Prognostic value of cytogenetics in adult patients with Philadelphia-negative acute lymphoblastic leukemia. *Ann Hematol* 2012, 91:19–25. 10.1007/s00277-011-1331-z 21935650

[3] Akagi T, Shih LY, Kato M, Kawamata N, Yamamoto G, Sanada M, et al: Hidden abnormalities and novel classification of t(15;17) acute promyelocytic leukemia (APL) based on genomic alterations. *Blood* 2009, 113:1741–1748. 10.1182/blood-2007-12-130260 19109227 PMC2647673

[4] Greif PA, Yaghmaie M, Konstandin NP, Ksienzyk B, Alimoghaddam K, Ghavamzadeh A, et al: Somatic mutations in acute promyelocytic leukemia (APL) identified by exome sequencing. *Leukemia* 2011, 25:1519–1522. 10.1038/leu.2011.114 21606962

[5] Welch JS, Westervelt P, Ding L, Larson DE, Klco JM, Kulkarni S, et al: Use of whole-genome sequencing to diagnose a cryptic fusion oncogene. *JAMA* 2011, 305:1577–1584. 10.1001/jama.2011.497 21505136 PMC3156695

[6] Cancer_Genome_Atlas_Research_Network: Genomic and epigenomic landscapes of adult de novo acute myeloid leukemia. *N Engl J Med* 2013, 368:2059–2074. 10.1056/NEJMoa1301689 23634996 PMC3767041

[7] Riva L, Ronchini C, Bodini M, Lo-Coco F, Lavorgna S, Ottone T, et al: Acute promyelocytic leukemias share cooperative mutations with other myeloid-leukemia subgroups. *Blood Cancer J* 2013, 3:e147 10.1038/bcj.2013.46 24036946 PMC3789210

[8] Chuang HY, Lee E, Liu YT, Lee D, Ideker T: Network-based classification of breast cancer metastasis. *Mol Syst Biol* 2007, 3:140 17940530 10.1038/msb4100180 PMC2063581

[9] Taylor IW, Linding R, Warde-Farley D, Liu Y, Pesquita C, Faria D, et al: Dynamic modularity in protein interaction networks predicts breast cancer outcome. *Nat Biotechnol* 2009, 27:199–204. 10.1038/nbt.1522 19182785

[10] Stoll G, Surdez D, Tirode F, Laud K, Barillot E, Zinovyev A, et al: Systems biology of Ewing sarcoma: a network model of EWS-FLI1 effect on proliferation and apoptosis. *Nucleic Acids Res* 2013, 41:8853–8871. 10.1093/nar/gkt678 23935076 PMC3799442

[11] Patel VN, Gokulrangan G, Chowdhury SA, Chen Y, Sloan AE, Koyuturk M, et al: Network signatures of survival in glioblastoma multiforme. PLoS Comput Biol 2013, 9:e1003237 10.1371/journal.pcbi.1003237 24068912 PMC3777929

[12] Minguez P, Gotz S, Montaner D, Al-Shahrour F, Dopazo J: SNOW, a web-based tool for the statistical analysis of protein-protein interaction networks. *Nucleic Acids Res* 2009, 37:W109–114. 10.1093/nar/gkp402 19454602 PMC2703972

[13] Mitra K, Carvunis AR, Ramesh SK, Ideker T: Integrative approaches for finding modular structure in biological networks. *Nat Rev Genet* 2013, 14:719–732. 10.1038/nrg3552 24045689 PMC3940161

[14] Durbin RM, Abecasis GR, Altshuler DL, Auton A, Brooks LD, Gibbs RA, et al: A map of human genome variation from population-scale sequencing. *Nature* 2010, 467:1061–1073. 10.1038/nature09534 20981092 PMC3042601

[15] Al-Shahrour F, Diaz-Uriarte R, Dopazo J: FatiGO: a web tool for finding significant associations of Gene Ontology terms with groups of genes. *Bioinformatics* 2004, 20:578–580. 14990455 10.1093/bioinformatics/btg455

[16] Alonso R, Salavert F, Garcia-Garcia F, Carbonell-Caballero J, Bleda M, Garcia-Alonso L, et al: Babelomics 5.0: functional interpretation for new generations of genomic data. *Nucleic Acids Res* 2015, 43:W117–121. 10.1093/nar/gkv384 25897133 PMC4489263

[17] Dolnik A, Engelmann JC, Scharfenberger-Schmeer M, Mauch J, Kelkenberg-Schade S, et al: Commonly altered genomic regions in acute myeloid leukemia are enriched for somatic mutations involved in chromatin remodeling and splicing. *Blood* 2012, 120:e83–92. 10.1182/blood-2011-12-401471 22976956

[18] Vogelstein B, Papadopoulos N, Velculescu VE, Zhou S, Diaz LA Jr., Kinzler KW: Cancer genome landscapes. *Science* 2013, 339:1546–1558. 10.1126/science.1235122 23539594 PMC3749880

[19] Goh KI, Cusick ME, Valle D, Childs B, Vidal M, Barabasi AL: The human disease network. *Proc Natl Acad Sci U S A* 2007, 104:8685–8690. 17502601 10.1073/pnas.0701361104 PMC1885563

[20] Oti M, Brunner HG: The modular nature of genetic diseases. *Clin Genet* 2007, 71:1–11. 17204041 10.1111/j.1399-0004.2006.00708.x

[21] Porta-Pardo E, Garcia-Alonso L, Hrabe T, Dopazo J, Godzik A: A Pan-Cancer Catalogue of Cancer Driver Protein Interaction Interfaces. *PLoS Comput Biol* 2015, 11:e1004518 10.1371/journal.pcbi.1004518 26485003 PMC4616621

[22] Gonzalez-Perez A, Perez-Llamas C, Deu-Pons J, Tamborero D, Schroeder MP, Jene-Sanz A, et al: IntOGen-mutations identifies cancer drivers across tumor types. *Nat Methods* 2013, 10:1081–1082. 10.1038/nmeth.2642 24037244 PMC5758042

[23] Garcia-Alonso L, Jimenez-Almazan J, Carbonell-Caballero J, Vela-Boza A, Santoyo-Lopez J, Antinolo G, Dopazo J: The role of the interactome in the maintenance of deleterious variability in human populations. *Mol Syst Biol* 2014, 10:752 10.15252/msb.20145222 25261458 PMC4299661

