# Supplementary figures and images for: The Mutational Landscape of Acute Promyelocytic Leukemia Reveals an Interacting Network of Co-Occurrences and Recurrent Mutations
Source: PLoS One. 2016 Feb 17;11(2):e0148346. doi: 10.1371/journal.pone.0148346 (PMC4757557; doi:10.1371/journal.pone.0148346)

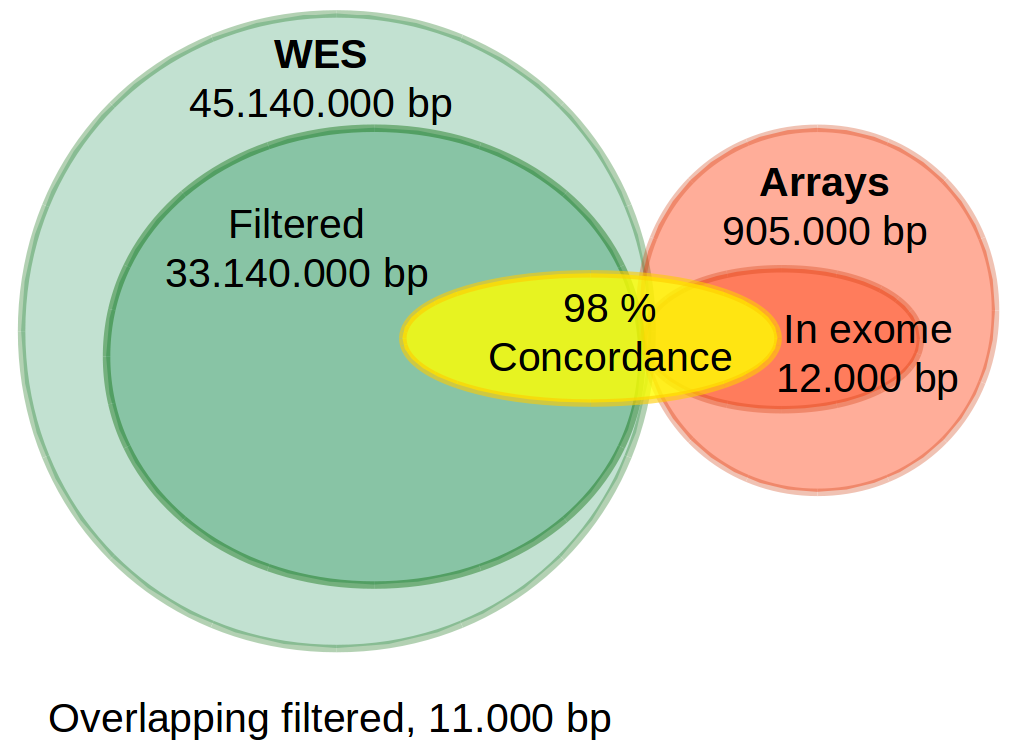

Supplement: S2 Fig — (TIF) [file pone.0148346.s002.tif]

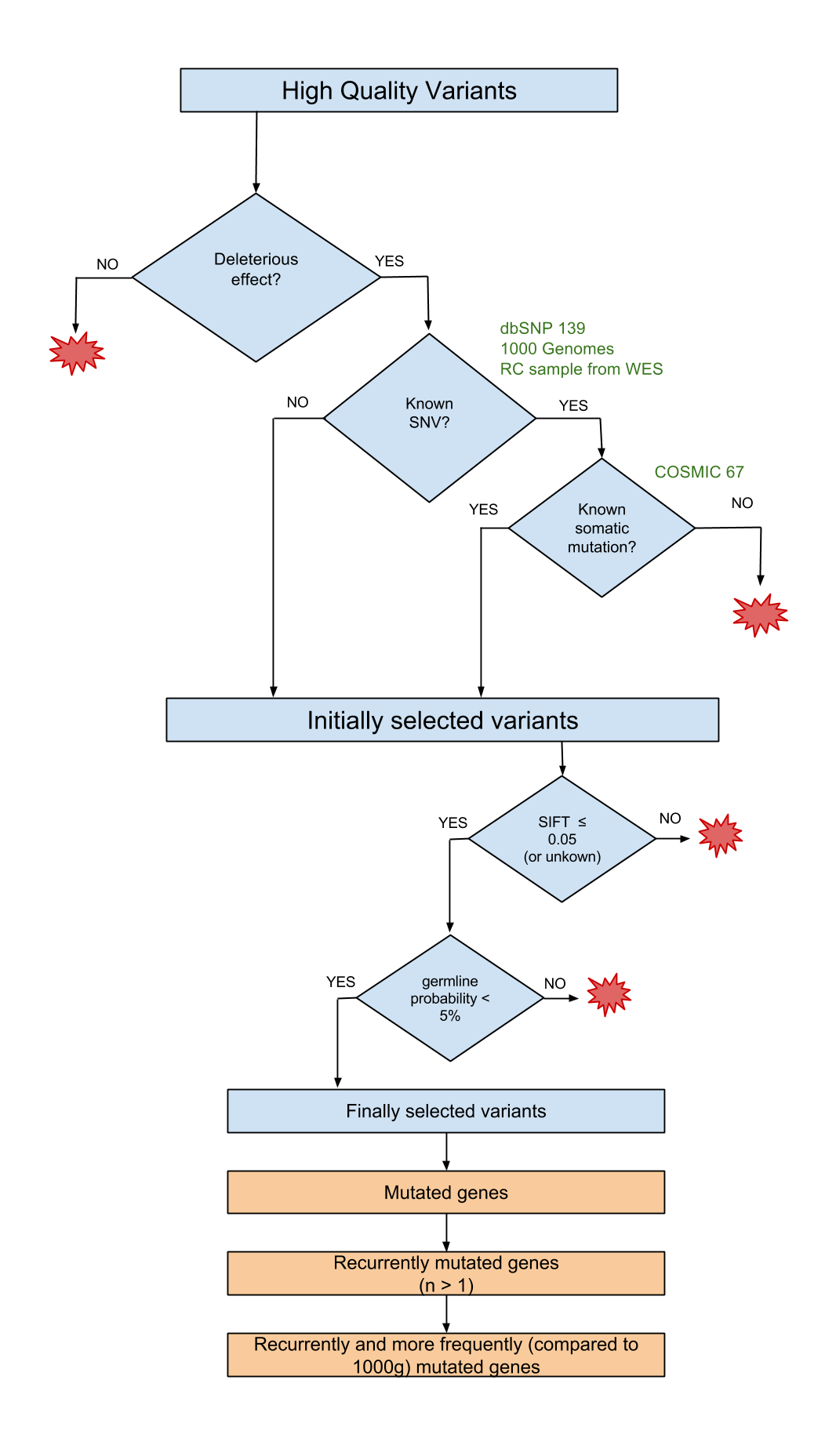

Supplement: S3 Fig — After trimming low quality reads and tails, PCR duplicated sequences were filtered. Selected reads, from DNA samples of APL diagnosis and completed remission cells, were aligned against human genome. A series of filters were applied for somatic mutation detection. (TIF) [file pone.0148346.s003.tif]

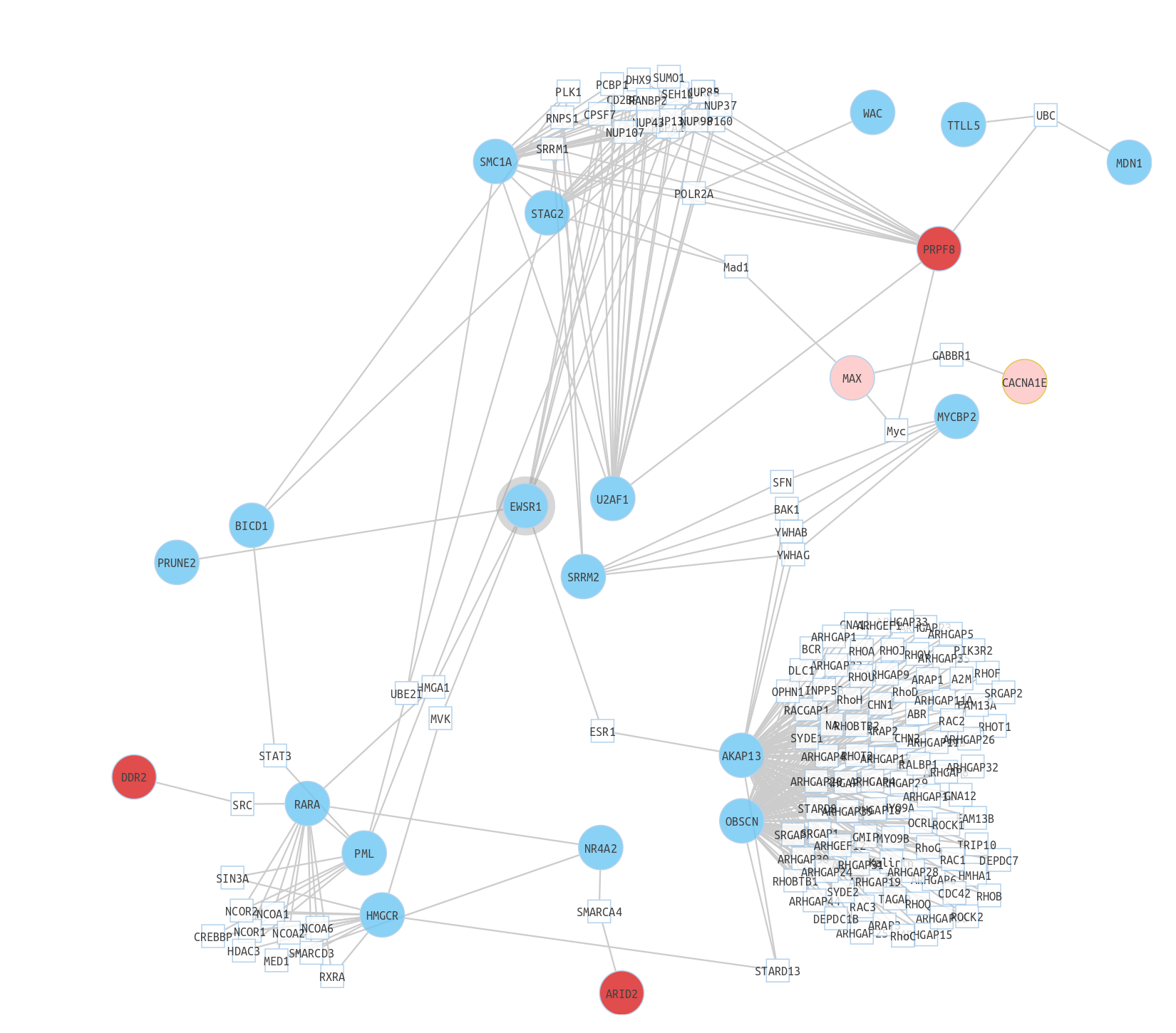

Supplement: S4 Fig — Genes are represented by nodes and their sizes defined from the number of significant co-occurrences they are implied. Edges represent co-occurrences between pairs of genes. Every edge is labelled with the number of samples that carries the mutated pair of genes as follows: higher than expected co-occurrences are coloured in green, while lower than expected (only one) in red. Edge width is proportional to the statistical p-value of chi-square test. Those genes co-occurring only at one single patient are painted in white. Seven co-occurrence subnetworks arise from the significant co-occurrence network, where remarkably a single component connected the half of represented genes. In contrast, 3 pairs are simultaneously mutated only in 2 different individuals, and 3 significant co-occurrence subnetworks, only in 1 patient. (TIF) [file pone.0148346.s004.tif]
