# Supplementary material for: The Mutational Landscape of Acute Promyelocytic Leukemia Reveals an Interacting Network of Co-Occurrences and Recurrent Mutations
Source: PLoS One. 2016 Feb 17;11(2):e0148346. doi: 10.1371/journal.pone.0148346 (PMC4757557; doi:10.1371/journal.pone.0148346)
Supplement: S1 Material and Methods — Table A. Main characteristics of the discovery cohort of patients. Table B. Primers used to validate mutations detected by WES (DOC) [file pone.0148346.s005.doc]

**The mutational landscape of Acute Promyelocytic Leukemia reveals an interacting network of co-occurrences and recurrent mutations**

Ibáñez, M.^1^*, Carbonell-Caballero, J.^2^*, García-Alonso, L.^2^, Such, E.^1^, Jiménez-Almazán, J.^2^, Vidal, E.^2^, Barragán, E.^3^, López-Pavía, M.^1^, LLop, M.^3^, Martín, I.^1^, Gómez-Seguí, I.^1^, Montesinos, P.^1^, Sanz, MA.^1^, Dopazo, J.^2,4,5+^, Cervera, J.^1,6+^

* Equal contribution

^+^ co-corresponding authors

^1^Hematology Service, Hospital Universitario y Politécnico La Fe, Valencia, Spain; ^2^Computational Genomics Department, Centro de Investigación Príncipe Felipe, Valencia, Spain; ^3^ Laboratory of Molecular Biology, Department of Clinical Chemistry, Hospital Universitario La Fe, Valencia, Spain. ^4^. Functional Genomics Node, Spanish National Institute of Bioinformatics at CIPF, 46012 Valencia, Spain. ^5^. Bioinformatics of Rare Diseases (BIER), CIBER de Enfermedades Raras (CIBERER), Valencia, Spain. ^6^ Genetics Unit, Hospital Universitario y Politécnico La Fe, Valencia, Spain

Running title:

The mutational landscape of APL

Corresponding authors:

José Vicente Cervera Zamora. Hospital Universitario La Fe. Avda. Fernando Abril Martorell, 106 CP.46026, Valencia, Phone: 961244688; E-mail: [cervera_jos@gva.es](mailto:cervera_jos@gva.es)

Joaquín Dopazo. Centro de Investigación Príncipe Felipe, C/ Eduardo Primo Yufera 3. 46012 Valencia, Spain Tel.: +34 96 328 96 80 / Fax: +34 96 328 97 01. E-mail: jdopazo@cipf.es

**S1 Material and Methods**

*Patients*

Samples were collected from patients diagnosed with *de novo* APL, defined by the presence of the *PML-RARA* rearrangement [^1^](#_ENREF_23). Available DNA sample at diagnosis was the only limiting criterion. All patients were enrolled in the consecutive multicenter PETHEMA LPA96, PETHEMA/HOVON LPA99 or LPA2005 trials between November 1998 and January 2013 and diagnosed at the Hospital Universitario y Politécnico La Fe of Valencia, Spain. Details about general exclusion and inclusion criteria, as well as treatment administration and follow-up have been reported elsewhere According to the Declaration of Helsinki, informed consent was obtained from all patients, and the protocol was approved by the Research Ethics Board.

Whole-exome sequencing (WES) was done on matched samples from 5 *de novo* APL patients, as our “discovery cohort” (Table A in S1 file). Diagnosis samples were obtained by bone marrow aspiration, and a corresponding complete molecular remission sample from bone marrow or peripheral blood was taken from each patient. Complete Remission (CR) was defined according to the recommendations of Cheson *et al*.^27^

Additionally, 25 APL patients with available DNA at diagnosis were selected for the extended analysis (“validation cohort”) to find recurrent mutations in the complete coding sequence of 97 genes (17 novel candidate genes from in-house results and 80 genes reported to be mutated in at least 1 patient from previous APL studies ). All samples were provided by the Hospital La Fe Biobank.

*DNA extraction and molecular analysis*

Genomic DNA (gDNA) was isolated using QIAmp DNA Blood Mini Kit extraction kits (QIAGEN GmbH Hilden, Germany) and UltraCleanTM extraction kits (MO BIO Carlsbad, CA, USA). The quality and concentration of DNA was assessed by a spectrophotometry with a Nano-Drop-1000 (NanoDrop Technologies Inc., Wilmington, DE) and integrity in an agarose gel. Detection of *FLT3-ITD* and D835 mutations was performed as previously described [^7^](#_ENREF_27).

*Construction of DNA libraries and sequencing data analysis*

Library preparation and exome capture were performed according to the protocol version 2.1 from Baylor College of Medicine and the sample preparation procedures for sequencing with SOLID 4 platform as recommended by the manufacturer.

*Sequenced reads processing pipeline*

Low quality reads were removed and low quality tails were trimmed (Supplementary Figure 1). PCR duplicated sequences were also filtered. Then, sequence reads were aligned to the reference human genome build GRCh37 (hg19) by using the SHRiMP tool[^8^](#_ENREF_28). Correctly mapped reads were evaluated (Samtools)[^9^](#_ENREF_29) and good quality mapped reads were realigned and recalibrated using GATK[^10^](#_ENREF_30). Individual variants (germline and somatic) were called using VarScan2[^11^](#_ENREF_31). The variants detected were filtered by germline and somatic p-values and low covered locus were discarded. Finally, somatic variants no present in the paired complete remission sample were selected. For the secondary analysis, only deleterious somatic variants were considered. Shared variants between individuals were detected to increase their association to disease. Intersection was done also at gene level. Finally, selected variants were annotated using both ANNOVAR[^12^](#_ENREF_32) and VARIANT[^13^](#_ENREF_33) with data on: presence in healthy population (dbSNP[^14^](#_ENREF_34) and 1000 genomes repository[^1^](#_ENREF_14)^5^), putative protein effect (SIFT[^16^](#_ENREF_35) and Polyphen[^17^](#_ENREF_36)), conservation (phastCons[^18^](#_ENREF_37)) and functional category (Gene Ontology[^19^](#_ENREF_38)). Variants were manually curated by *de visu* confirmation using IGV software^20^.

*Single nucleotide polymorphisms array (SNP­-A) analyses*

To check the quality of our pipeline analysis, we compared the results from WES and SNP-A 6.0 arrays (Affymetrix, Santa Clara, CA). Technical details regarding sample processing for SNP-A assays have been reported elsewhere[^2^](#_ENREF_2)^1^.

*Validated mutations of candidate genes*

To confirm candidate variants (point and indel mutations) we sequenced both samples of each patient by conventional Sanger methods. We performed a PCR amplification and direct DNA sequencing using specific primers (Table B in S1 file). The PCR products were visualized, purified, and sequenced according to standard methodologies, using an ABIPRISM 3130 DNA Analyzer (Applied Biosystems, Foster City, CA). Sequence traces were analyzed using Applied Biosystems software and reviewed manually. The analysis was checked by its corresponding GeneBank Accession Number.

*Extended analysis*

Targeted resequencing using Haloplex technology and sequence analysis

The Haloplex target enrichment system (Agilent) was performed on an extended cohort (n=25) of patients. We designed a custom panel of 97 genes (Supplementary Table 5) using SureDesign Tool (Agilent) for next-generation sequencing, according to the manufacturer’s instructions[^22^](#_ENREF_41). Seventeen candidate genes were selected from in-house results and 80 genes from previously published APL series. This panel was composed of genes from 10 different categories: signaling genes (20 genes), transcription factors (12 genes), tumor suppressor (4 genes), splicing machinery (5 genes), protein kinase activity (10 genes) and ubiquitination (3 genes), transmembrane proteins (12 genes), metabolism (9 genes) and other cell function (21 genes). The enriched and barcoded targets were deep-sequenced on an Illumina next generation sequencing platform[^23^](#_ENREF_42). Selected variants were annotated as described in Suplementary Figure 2.

Next-generation sequencing data from other APL series

We compared our results with other APL series, namely APL cases from The Cancer Genome Atlas Network (TCGA)[^6^](#_ENREF_6), with publicly available data (n=20), and the series from Greif *et al*. [^2^](#_ENREF_4) (n=3) and Riva *et al.* [^24^](#_ENREF_7) (n=11).

Network analysis

Network analysis of the candidate gene products was carried out by finding significant subnetworks with the SNOW [^25^](#_ENREF_13) tool, as implemented in the Babelomics [^26^](#_ENREF_16) web package (available at: www.babelomics.org). SNOW maps the candidate genes onto the human interactome, calculates the Minimal Connected Network (MCN) (the smallest subnetwork that connects all the genes in the list) allowing one intermediate interaction. Then, it evaluates whether the subnetwork found is larger and more connected that the random expectation by comparing the subnetwork versus empirical MCNs generated from 1,000 random same-size gene lists.

*Statistical methods*

Software R 2.13.0 was used in the statistical analysis. Descriptive and comparative analysis was performed (absolute and relative frequencies; mean/standard deviation; median/interval; Chi-squared test; Mann-Whitney U Test, ANOVA). Unadjusted time-to-event analysis were performed using the Kaplan-Meier estimate and for comparisons, long-rank tests. Last update on clinical data was performed on April 2014. All *P* values reported are two-sided. Complete Remission (CR) and resistant disease were defined according to the recommendations of Cheson et al.^27^ Overall survival (OS) was measured from the time of diagnosis to the time of last follow-up or death from any cause. Disease-free survival (DFS) and relapse-free survival (RFS) were measured from the date of CR. In the analysis of DFS, relapse or death, whichever occurred first, was considered an uncensored event. For RFS, relapse was considered an uncensored event.

We implemented a germinality test based on a binomial model. Here, somatic expectation is compared against the probability of the three possible germline genotypes derived from the observed alternative allele read proportion, being, at the end, only selected those variants exhibiting a germline probability under a 5%.

**References**

1 Bolufer, P., Colomer, D., Gomez, M. T. et al. Quantitative assessment of PML-RARa and BCR-ABL by two real-time PCR instruments: multiinstitutional laboratory trial. *Clin Chem* 2004; 50, 1088-1092.

2 Greif, P. A., Yaghmaie, M., Konstandin, N. P. et al. Somatic mutations in acute promyelocytic leukemia (APL) identified by exome sequencing. *Leukemia* 2011; 25, 1519-1522.

3 Sanz, M. A., Martin, G., Rayon, C. et al. A modified AIDA protocol with anthracycline-based consolidation results in high antileukemic efficacy and reduced toxicity in newly diagnosed PML/RARalpha-positive acute promyelocytic leukemia. PETHEMA group. *Blood* 1999; 94, 3015-3021.

4 Sanz, M. A., Martin, G., Gonzalez, M. et al. Risk-adapted treatment of acute promyelocytic leukemia with all-trans-retinoic acid and anthracycline monochemotherapy: a multicenter study by the PETHEMA group. *Blood* 2004; 103, 1237-1243.

5 Sanz, M. A., Montesinos, P., Vellenga, E. et al. Risk-adapted treatment of acute promyelocytic leukemia with all-trans retinoic acid and anthracycline monochemotherapy: long-term outcome of the LPA 99 multicenter study by the PETHEMA Group. *Blood* 2008; 112, 3130-3134.

6 Cancer_Genome_Atlas_Research_Network. Genomic and epigenomic landscapes of adult de novo acute myeloid leukemia. *N Engl J Med* 2013; 368, 2059-2074.

7 Luna, I., Such, E., Cervera, J. et al. Analysis of SNP rs16754 of WT1 gene in a series of de novo acute myeloid leukemia patients. *Ann Hematol* 2012; 91, 1845-1853.

8 Rumble, S. M., Lacroute, P., Dalca, A. V. et al. SHRiMP: accurate mapping of short color-space reads. *PLoS Comput Biol* 2009; 5, e1000386.

9 Li, H., Handsaker, B., Wysoker, A. et al. The Sequence Alignment/Map format and SAMtools. *Bioinformatics* 2009; 25, 2078-2079.

10 McKenna, A., Hanna, M., Banks, E. et al. The Genome Analysis Toolkit: a MapReduce framework for analyzing next-generation DNA sequencing data. *Genome Res* 2010; 20, 1297-1303.

11 Koboldt, D. C., Zhang, Q., Larson, D. E. et al. VarScan 2: somatic mutation and copy number alteration discovery in cancer by exome sequencing. *Genome Res* 2012; 22, 568-576.

12 Wang, K., Li, M. & Hakonarson, H. ANNOVAR: functional annotation of genetic variants from high-throughput sequencing data. *Nucleic Acids Res* 2010; 38, e164.

13 Medina, I., De Maria, A., Bleda, M. et al. VARIANT: Command Line, Web service and Web interface for fast and accurate functional characterization of variants found by Next-Generation Sequencing. *Nucleic Acids Res* 2012; 40, W54-58.

14 Sherry, S. T., Ward, M. H., Kholodov, M. et al. dbSNP: the NCBI database of genetic variation. *Nucleic Acids Res* 2001; 29, 308-311.

15 Durbin, R. M., Abecasis, G. R., Altshuler, D. L. et al. A map of human genome variation from population-scale sequencing. *Nature* 2010; 467, 1061-1073.

16 Kumar, P., Henikoff, S. & Ng, P. C. Predicting the effects of coding non-synonymous variants on protein function using the SIFT algorithm. *Nat Protoc* 2009; 4, 1073-1081.

17 Adzhubei, I. A., Schmidt, S., Peshkin, L. et al. A method and server for predicting damaging missense mutations. *Nat Methods* 2010; 7, 248-249.

18 Siepel, A., Bejerano, G., Pedersen, J. S. et al. Evolutionarily conserved elements in vertebrate, insect, worm, and yeast genomes. *Genome Res* 2005; 15, 1034-1050.

19 Ashburner, M., Ball, C. A., Blake, J. A. et al. Gene ontology: tool for the unification of biology. The Gene Ontology Consortium. *Nat Genet* 2000; 25, 25-29.

20 Robinson, J. T., Thorvaldsdottir, H., Winckler, W. et al. Integrative genomics viewer. *Nat Biotechnol* 2011; 29, 24-26.

21 Gomez-Segui, I., Cervera, J., Such, E. et al. Prognostic value of cytogenetics in adult patients with Philadelphia-negative acute lymphoblastic leukemia. *Ann Hematol* 2012; 91, 19-25.

22 Dahl, F., Stenberg, J., Fredriksson, S. et al. Multigene amplification and massively parallel sequencing for cancer mutation discovery. *Proc Natl Acad Sci U S A* 2007; 104, 9387-9392.

23 Bentley, D. R., Balasubramanian, S., Swerdlow, H. P. et al. Accurate whole human genome sequencing using reversible terminator chemistry. *Nature* 2008; 456, 53-59.

24 Riva, L., Ronchini, C., Bodini, M. et al. Acute promyelocytic leukemias share cooperative mutations with other myeloid-leukemia subgroups. *Blood cancer journal* 2013; 3, e147.

25 Minguez, P., Gotz, S., Montaner, D., Al-Shahrour, F. & Dopazo, J. SNOW, a web-based tool for the statistical analysis of protein-protein interaction networks. *Nucleic Acids Res* 2009; 37, W109-114.

26 Medina, I., Carbonell, J., Pulido, L. et al. Babelomics: an integrative platform for the analysis of transcriptomics, proteomics and genomic data with advanced functional profiling. *Nucleic Acids Res* 2010; 38, W210-213

27 Cheson BD, Bennett JM, Kopecky KJ, et al.; International Working Group for Diagnosis, Standardization of Response Criteria, Treatment Outcomes, and Reporting Standards for Therapeutic Trials in Acute Myeloid Leukemia. Revised recommendations of the International Working Group for Diagnosis, Standardization of Response Criteria, Treatment Outcomes, and Reporting Standards for Therapeutic Trials in Acute Myeloid Leukemia. J Clin Oncol 2003;21 24: 4642–9.

**Supplementary Material and Methods Tables.**

**Table A. Main characteristics of the discovery cohort of patients.**

| **Patient** | ***APL_1*** | ***APL_2*** | ***APL_3*** | ***APL_4*** | ***APL_5*** |
| --- | --- | --- | --- | --- | --- |
| FAB | M3 | M3-v | M3 | M3 | M3-v |
| Age at Dg | 73 | 34 | 37 | 39 | 43 |
| Sex | M | F | M | M | M |
| Protocol | 96 | 99 | 99 | 99 | 99 |
| WGS consent | Yes | Yes | Yes | Yes | Yes |
| %BM Blast | 84 | 93 | 76 | 98 | 95 |
| WBC | 1 | 23,7 | 3,3 | 7,9 | 15,4 |
| Clinical Cytogenetics | t(15;17), +8 | t(15;17) | t(15;17) | NA. PML/RARA positive | t(15;17) |
| FLT3-ITD | Negative | Positive | Negative | Negative | Positive |
| Relapse | No | No | No | No | No |
| Expired | No | No | No | No | No |
| OS (mo) | 122 | 54 | 108 | 101 | 91 |
| Deleterious mutations (SNVs+indels) | 14 | 7 | 10 | 8 | 11 |

M, male; F, female: NA, not available

**Table B.** Primers used to validate mutations detected by WES

| Gene | Forward (5´🡪3´) | Reverse (5´🡪3´) | Pair bases |
| --- | --- | --- | --- |
| *ADC* | GGATTGGACAGGACTTGAAGG | CCCTGGGGAAGGTCTCATA | 396 |
| *ALPK3* | AGGCAACCACACCTGAAGAA | TCTGTCATGGGGTAGTGCTG | 374 |
| *APPL1* | GGATCGTAGGGCATCAGAAA | AAAATGGCTTTCAGCAATGG | 403 |
| *AQP6* | CCTGGCCTCACTGATCTACA | AGGCGTAGGCTGTTTCACAC | 189 |
| *ATP2A3* | ATGATCACGGGGGATAACAA | GGCCTCACCATAGCAGTGAT | 242 |
| *ATXN3* | TCAGACTAACTGCTCTTGCATTC | TCCCAAAGTGCTGGGATTAC | 154 |
| *BRCA* | TGTGTTGAAATTGTAAATACCTTGG | TTCTGCCTTTTGGCTAGGTG | 201 |
| *C12orf35* | AATGCCCACTGTGATGTGAA | CACTTTCCAAAATGCGTCCT | 239 |
| *C14orf106* | CATGGCCATCAATCAGAAGA | CGTTCATCGCAATCTTCCTT | 231 |
| *C1orf129* | TGAGCATGTTTTCCAGTCTGA | CACCAAATTATGATGGTGAAGTTT | 333 |
| *CCDC142* | ACCCTCTTCTCCCAAACACTC | CCTGCCACTTCCACCTTTAC | 281 |
| *CCDC27* | TGGGTGCCACACATACTCAG | ACAGACCCCGGTTCTACCTC | 378 |
| *CRIP3* | ACCACTTCTTTCCACCACCA | GGTTCAGGCCTAGAGGGAAC | 412 |
| *CSNK1A1L* | CCTCCATCTTTGCCTCTCAG | CCCACCTTGAAGAATCGTGT | 231 |
| *DACH2* | TTCCCAGAGGCAGCTTACAT | CTGCCAGCCAAAGGTTTCTA | 425 |
| *DTNA* | GGTGCCAACGAAACTACAGC | TGGAAGGAAGAAGGCTCTGA | 321 |
| *EPB41L4A* | AGAGCAGCTTGGTTTCTGATG | AGTGGCGCACACAACCTT | 263 |
| *ETNK1* | CATGCAGTATTCCAGCCAAA | GACGAAAATTTAGAAGCATTTTCA | 357 |
| *FAM171A1* | TAACCTGAGGGAGGTGGAGA | TGGACATGGAAGGTTTGTCA | 364 |
| *FAM48B2* | GAATCGAAAACCCGCATCCGA | TCGCCCATGCAACAAAATTGCA | 357 |
| *FBLN1* | TAACCTGAGGGAGGTGGAGA | TGGACATGGAAGGTTTGTCA | 364 |
| *FILIP1L* | CGTTTCCTGAGCTCTTCCAC | CCTTGCTGAAGCCAGAGTTC | 309 |
| *FLT3* | TCCATCACCGGTACCTCCTA | CCTGAAGCTGCAGAAAAACC | 392 |
| *FLT3* | GGCTGGTCTTGAACTCCTTG | TTCATGAAAGAGTCAATAGGTCAGA | 302 |
| *GJB7* | CCACACCTTCACTTCTGGTG | GGGACAAGGCTTCAAATCAC | 236 |
| *GPRIN2* | CTAGACCTGGGGGACACAAC | GACGCCACTAGTTTGGGAAA | 197 |
| *HMGCR* | TGGAGGACAGGGCTTAAATG | ACATTCTGTGCTGCATCCTG | 373 |
| *KIAA0317* | AAACATCCAGGCAGATGTCA | CAAAAGGTTCTCAGGGACCA | 326 |
| *KIF14* | AGGAATTGTTTTGCCTAATGG | CTGGGCAACAGAGCAAGACT | 284 |
| *MDN1* | TGTGGCTTTTTGGGGTTTTAC | CTGTCACCCCCCAGAAAAGAA | 289 |
| *MSR1* | AACCTGAACTCCGAGGGTTT | CCAGGCTACTGGTTATCGTACC | 461 |
| *NCL* | GCTGGGAACCACTGTTTCAG | TGGCTTTTCTGACAGGATCA | 268 |
| *NR4A2* | TACGACCCATTTGGAGGAAG | TTACCCCCGTTGAATCTGAG | 458 |
| *ORC3* | GTTACATTAGCTGTATTGATGGTAAG | TGCTGCAGAAACATATCCTTG | 327 |
| *PPP1R16B* | TGGAGCAGCTACAAGGAACA | GAAGTTCTGCCTCCGATCAA | 173 |
| *PRICKLE2* | ATGTGTTTCACGTCCCAGTG | GCTGACAGCCGGTTAGAACT | 383 |
| *RECQL5* | GTGAGGGGAGGCACTCAGTG | GCCCGTCTCTTACCTTCACA | 391 |
| *RFK* | GGAAAAACTGCACTTAGATTTTAACC | ATGGGGAAATCCTCAATGTG | 488 |
| *ROCK1* | ATATGTTTCCCCTATGAAAATGTTG | GCTGTGTCCGATTCTGTCCT | 294 |
| *SARNP* | GAGATCACAAGCAAGAAGTGTCA | GCCCAGGAAGGCCTAAGTAT | 302 |
| *SLC35A5* | GGGCCTTCAGAGGAGTAACC | GGGCTTCCAAGAAAAATTCC | 242 |
| *SULT6B1* | CACATCAGGGAATGGTTGTAGA | GAATCTCCAAACCGGAAGAA | 294 |
| *TFPI2* | CCCACAAAATGTTGCTGAAA | TCCACCCAAATGTCTTTTCA | 334 |
| *TIAM2* | AGGGAACCTTGCTGGACTCT | GCTGCTCTCCTCCTTTCTGA | 339 |
| *TMBIM4* | ACTTGCCTTGCCTTTTTCTG | TGCATCAGTGAGTGTGTGTCA | 254 |
| *TPRT* | GGGATGCTGAAATAATATGTGC | CAGACACTTGGTTGGTGCAG | 243 |
| *TTN* | AAATTGTTTCACAGCTAACATGG | TCTGGATGCTGGTTAGGTCA | 463 |
| *UBR2* | TCTGCTATTGCTTAACTCTCATGC | ACAAAAGGCCCACCCATTAT | 330 |
| *USP6* | CAGAAGCAATCAAGTGGGTGT | GCACTTTAATAGTTGAAAGAAGAAAAA | 216 |
| *ZNF518B* | GGAACTTTAAGCCAGGCAAA | ATGCCTGTGTGTTTCACCAA | 289 |
