# Supplementary material for: The Mutational Landscape of Acute Promyelocytic Leukemia Reveals an Interacting Network of Co-Occurrences and Recurrent Mutations
Source: PLoS One. 2016 Feb 17;11(2):e0148346. doi: 10.1371/journal.pone.0148346 (PMC4757557; doi:10.1371/journal.pone.0148346)
Supplement: S1 Table — (DOCX) [file pone.0148346.s006.docx]

**Supplementary tables**

**Supplementary table 1.** Sequencing results obtained for the samples sequenced

| **sample** | **raw reads** | **filtered reads** | **mapped reads** | **mean coverage on target** | **% of target with a coverage > 15x** | **% of target with a coverage > 10x** |
| --- | --- | --- | --- | --- | --- | --- |
| APL_1_Dx | 176222401 | 109184284 | 92982362 | 67.2X | 75.1% | 80.5% |
| APL_1_CR | 144681613 | 97591715 | 81607693 | 57.6X | 75,0% | 80.2% |
| APL_2_Dx | 180920506 | 111582286 | 95209796 | 68.0X | 76.3% | 81.3% |
| APL_2_CR | 198815649 | 96331158 | 85221060 | 59.3X | 75.1% | 81,0% |
| APL_3_Dx | 165553521 | 101357335 | 86210433 | 57.3X | 72.6% | 78.4% |
| APL_3_CR | 178063640 | 96436716 | 89519030 | 62.8X | 74.3% | 79.9% |
| APL_4_Dx | 167917831 | 77912798 | 81014437 | 55.7X | 76.1% | 82.1% |
| APL_4_CR | 163390096 | 94902832 | 81452258 | 57.1X | 74.9% | 81,0% |
| APL_5_Dx | 174929724 | 103657417 | 88752599 | 63.0X | 75.7% | 81.2% |
| APL_5_CR | 183434386 | 110986238 | 94733472 | 66.7X | 75.3% | 80.6% |
| Total | 173392937 | 99994277.9 | 87670314 | 61.5X | 75.1% | 81,0% |
