# Supplementary material for: The Mutational Landscape of Acute Promyelocytic Leukemia Reveals an Interacting Network of Co-Occurrences and Recurrent Mutations
Source: PLoS One. 2016 Feb 17;11(2):e0148346. doi: 10.1371/journal.pone.0148346 (PMC4757557; doi:10.1371/journal.pone.0148346)
Supplement: S2 Table — (DOCX) [file pone.0148346.s007.docx]

**Supplementary tables**

**Supplementary table 2**. Mutations detected by whole exome sequencing in the study cohort.

| **Group** | **Annotated Gene** | **Annotated Transcript** | **CHR** | **Start Position** | **End position** | **Ref.** | **Variant** | **Codon Change** | **AA Change** | **Validation** | **Type** | **Sift** | **Polyphen** | **Consequence Type** | **Cons.** | **Total reads** | **Reference reads** | **Variant reads** | **VarFreqAllel** | **dbSNP ID** | **1000 genome project allele frequency** | **ESP project allele frequency** |
| --- | --- | --- | --- | --- | --- | --- | --- | --- | --- | --- | --- | --- | --- | --- | --- | --- | --- | --- | --- | --- | --- | --- |
| APL_1 | TMBIM4 | NM_016056 | 12 | 66531936 | 66531937 | TA | T | c.520delT | p.Y174fs | Both samples | DEL | unknown | unknown | frameshift deletion | 300 | 22 | 15 | 7 | 32 | unknown | unknown | unknown |
| APL_1 | ADC | NM_052998 | 1 | 33558894 | 33558894 | G | A | c.G464A | p.C155Y | 1 | SNV | deleterious (0,06) | deleterious (0,998) | nonsynonymous SNV | 478 | 101 | 60 | 41 | 41 | unknown | unknown | unknown |
| APL_1 | HMGCR | NM_000859 | 5 | 74655086 | 74655086 | A | T | c.A2249T | p.N750I | 1 | SNV | deleterious (0) | 1,00 | nonsynonymous SNV | 460 | 79 | 47 | 32 | 41 | unknown | unknown | unknown |
| APL_1 | ORC3 | NM_181837 | 6 | 88317429 | 88317429 | A | G | c.A466G | p.M156V | 1 | SNV | deleterious (0,06) | 0,28 | nonsynonymous SNV | 363 | 92 | 57 | 35 | 38 | unknown | unknown | unknown |
| APL_1 | PRICKLE2 | NM_198859 | 3 | 64133212 | 64133213 | AC | A | c.953delG | p.G318fs | 1 | DEL | unknown | unknown | frameshift deletion | 479 | 29 | 12 | 17 | 59 | unknown | unknown | unknown |
| APL_1 | ZNF518B | NM_053042 | 4 | 10447523 | 10447523 | T | C | c.A430G | p.T144A | 1 | SNV | deleterious (0) | 0,40 | nonsynonymous SNV | 374 | 138 | 92 | 46 | 33 | unknown | unknown | unknown |
| APL_1 | ATP2A3 | NM_174958 | 17 | 3844342 | 3844354 | AGCGGGCGGTGCG | A | c.2011_2022del | p.671_674del | 0 | DEL | unknown | unknown | nonframeshift deletion | 445 | 20 | 12 | 8 | 40 | unknown | unknown | unknown |
| APL_1 | BRCA2 | NM_000059 | 13 | 32911713 | 32911713 | G | A | c.G3221A | p.S1074N | 0 | SNV | deleterious (0,3) | 0,02 | nonsynonymous SNV | 0 | 90 | 55 | 35 | 39 | unknown | unknown | unknown |
| APL_1 | KIF14 | NM_014875 | 1 | 200550331 | 200550332 | GC | G | c.3332delG | p.G1111fs | 0 | DEL | unknown | unknown | frameshift deletion | 461 | 33 | 25 | 8 | 24 | unknown | unknown | unknown |
| APL_1 | MSR1 | NM_002445 | 8 | 16021659 | 16021662 | CACT | C | c.729_731del | p.243_244del | 0 | DEL | unknown | unknown | nonframeshift deletion | 443 | 448 | 256 | 192 | 43 | unknown | unknown | unknown |
| APL_1 | NCL | NM_005381 | 2 | 232321713 | 232321714 | CT | C | c.1569delA | p.K523fs | 0 | DEL | unknown | unknown | frameshift deletion | 591 | 124 | 84 | 40 | 32 | unknown | unknown | unknown |
| APL_1 | TFPI2 | NM_006528 | 7 | 93518409 | 93518409 | T | C | c.A398G | p.N133S | 0 | SNV | deleterious (0,29) | 1,00 | nonsynonymous SNV | 0 | 26 | 20 | 6 | 23 | unknown | unknown | unknown |
| APL_1 | TTN | NM_133378 | 2 | 179570017 | 179570017 | C | T | c.G25756A | p.V8586I | 0 | SNV | unknown | unknown | nonsynonymous SNV | 666 | 127 | 89 | 38 | 30 | unknown | unknown | unknown |
| APL_1 | UBR2 | NM_015255 | 6 | 42647530 | 42647530 | T | C | c.T4678C | p.F1560L | 0 | SNV | deleterious (0,1) | 0,12 | nonsynonymous SNV | 633 | 191 | 86 | 105 | 55 | unknown | unknown | unknown |
| APL_2 | SULT6B1 | NM_001032377 | 2 | 37414527 | 37414527 | G | A | c.C169T | p.L57F | Both samples | SNV | deleterious (0,01) | 0,19 | nonsynonymous SNV | 517 | 49 | 32 | 17 | 35 | unknown | unknown | unknown |
| APL_2 | FLT3 | NM_004119 | 13 | 28610141 | 28610141 | G | A | c.C1349T | p.A450V | 1 | SNV | benign (1) | 0,00 | nonsynonymous SNV | 391 | 97 | 59 | 38 | 39 | rs144444671 | unknown | 0,000 |
| APL_2 | GJB7 | NM_198568 | 6 | 87994263 | 87994263 | G | T | c.C368A | p.A123D | 1 | SNV | deleterious (0) | 0,00 | nonsynonymous SNV | 422 | 79 | 42 | 37 | 47 | unknown | unknown | unknown |
| APL_2 | DTNA | NM_001198944 | 18 | 32462130 | 32462130 | T | G | c.T1135G | p.Y379D | 0 | SNV | deleterious (0) | 0,98 | nonsynonymous SNV | 586 | 34 | 26 | 8 | 24 | unknown | unknown | unknown |
| APL_2 | EPB41L4A | NM_022140 | 5 | 111500816 | 111500816 | C | CTAAAA | c.1933-1G>TTTTAG | - | 0 | INS | unknown | unknown | splicing | 517 | 66 | 44 | 22 | 33 | unknown | unknown | unknown |
| APL_2 | FAM48B2 | NM_001136233 | X | 24330069 | 24330069 | C | G | c.G1364C | p.G455A | 0 | SNV | unknown | unknown | nonsynonymous SNV | 0 | 14 | 7 | 7 | 50 | unknown | unknown | unknown |
| APL_2 | USP6 | NM_004505 | 17 | 5044723 | 5044723 | A | G | c.A1502G | p.H501R | 0 | SNV | benign (1) | 0,05 | nonsynonymous SNV | 597 | 21 | 15 | 6 | 29 | unknown | unknown | unknown |
| APL_3 | ATXN3 | NM_001164781 | 14 | 92537353 | 92537356 | CCCT | C | c.704_706del | p.235_236del | Both samples | DEL | unknown | unknown | nonframeshift deletion | 435 | 38 | 22 | 16 | 42 | unknown | unknown | unknown |
| APL_3 | ALPK3 | NM_020778 | 15 | 85401766 | 85401766 | A | G | c.A4403G | p.K1468R | 1 | SNV | deleterious (0) | 0,96 | nonsynonymous SNV | 333 | 38 | 20 | 18 | 47 | unknown | unknown | unknown |
| APL_3 | CSNK1A1L | NM_145203 | 13 | 37679273 | 37679273 | C | T | c.G121A | p.E41K | 1 | SNV | deleterious (0,02) | 0,90 | nonsynonymous SNV | 827 | 141 | 98 | 43 | 30 | unknown | unknown | unknown |
| APL_3 | FILIP1L | NM_182909 | 3 | 99569571 | 99569571 | C | T | c.G949A | p.D317N | 1 | SNV | deleterious (0,01) | 0,97 | nonsynonymous SNV | 684 | 172 | 112 | 60 | 35 | unknown | unknown | unknown |
| APL_3 | FLT3 | NM_004119 | 13 | 28592641 | 28592641 | T | A | c.A2504T | p.D835V | 1 | SNV | deleterious (0) | 1,00 | nonsynonymous SNV | 440 | 109 | 86 | 23 | 21 | rs121909646 | unknown | unknown |
| APL_3 | C12orf35 | NM_018169 | 12 | 32134637 | 32134637 | C | A | c.C748A | p.L250I | 0 | SNV | deleterious (0,34) | 0,30 | nonsynonymous SNV | 0 | 31 | 23 | 8 | 26 | unknown | unknown | unknown |
| APL_3 | CCDC27 | NM_152492 | 1 | 3683876 | 3683877 | AG | A | c.1611delG | p.Q537fs | 0 | DEL | unknown | unknown | frameshift deletion | 251 | 25 | 17 | 8 | 32 | unknown | unknown | unknown |
| APL_3 | PPP1R16B | NM_015568 | 20 | 37547141 | 37547141 | T | TC | c.1536_1537insC | p.S512fs | 0 | INS | unknown | unknown | frameshift insertion | 571 | 17 | 13 | 4 | 24 | unknown | unknown | unknown |
| APL_3 | SLC35A5 | NM_017945 | 3 | 112299969 | 112299969 | G | A | c.G1005A | p.M335I | 0 | SNV | unknown | 0,97 | nonsynonymous SNV | 622 | 59 | 44 | 15 | 25 | unknown | unknown | unknown |
| APL_3 | TIAM2 | NM_012454 | 6 | 155577812 | 155577812 | G | C | c.G4663C | p.A1555P | 0 | SNV | deleterious (0,02) | 0,06 | nonsynonymous SNV | 0 | 30 | 23 | 7 | 23 | unknown | 0,001 | unknown |
| APL_4 | FBLN1 | NM_006487 | 22 | 45943051 | 45943051 | C | T | c.C1408T | p.Q470X | 1 | SNV | deleterious (0,01) | 0,72 | stopgain SNV | 561 | 62 | 38 | 24 | 39 | unknown | unknown | unknown |
| APL_4 | KIAA0317 | NM_001039479 | 14 | 75134254 | 75134254 | T | C | c.A1958G | p.N653S | 1 | SNV | deleterious (0,02) | 0,99 | nonsynonymous SNV | 576 | 69 | 36 | 33 | 48 | unknown | unknown | unknown |
| APL_4 | MDN1 | NM_014611 | 6 | 90503818 | 90503818 | C | T | c.662+1G>A | - | 1 | SNV | unknown | unknown | splicing | 483 | 36 | 24 | 12 | 33 | unknown | unknown | unknown |
| APL_4 | PTPRT | NM_133170 | 20 | 40864875 | 40864875 | T | C | c.A2393G | p.Y798C | 1 | SNV | deleterious (0,02) | 0,84 | nonsynonymous SNV | 676 | 48 | 27 | 21 | 44 | unknown | unknown | unknown |
| APL_4 | AQP6 | NM_001652 | 12 | 50369382 | 50369383 | AG | A | c.778delG | p.G260fs | 0 | DEL | unknown | unknown | frameshift deletion | 0 | 17 | 10 | 7 | 41 | unknown | unknown | unknown |
| APL_4 | C14orf106 | NM_018353 | 14 | 45693721 | 45693722 | CT | C | c.2068delA | p.S690fs | 0 | DEL | unknown | unknown | frameshift deletion | 251 | 17 | 12 | 5 | 29 | unknown | unknown | unknown |
| APL_4 | RECQL5 | NM_004259 | 17 | 73626918 | 73626918 | C | CTG | c.1586-1G>CAG | - | 0 | INS | unknown | unknown | splicing | 361 | 32 | 15 | 17 | 53 | unknown | unknown | unknown |
| APL_4 | SARNP | NM_033082 | 12 | 56188152 | 56188153 | GA | G | c.396delT | p.V132fs | 0 | DEL | unknown | unknown | frameshift deletion | 612 | 16 | 4 | 12 | 75 | unknown | unknown | unknown |
| APL_5 | C1orf129 | NM_025063 | 1 | 170927607 | 170927608 | TA | T | c.79delA | p.K27fs | Both samples | DEL | unknown | unknown | frameshift deletion | 0 | 10 | 4 | 6 | 60 | unknown | unknown | unknown |
| APL_5 | APPL1 | NM_012096 | 3 | 57302516 | 57302516 | G | A | c.1983+1G>A | - | 1 | SNV | unknown | unknown | splicing | 567 | 66 | 52 | 14 | 21 | unknown | unknown | unknown |
| APL_5 | FAM171A1 | NM_001010924 | 10 | 15258058 | 15258058 | G | A | c.C923T | p.A308V | 1 | SNV | deleterious (0,14) | 0 | nonsynonymous SNV | 662 | 102 | 78 | 24 | 24 | unknown | unknown | unknown |
| APL_5 | NR4A2 | NM_006186 | 2 | 157184370 | 157184370 | T | G | c.A1151C | p.Y384S | 1 | SNV | deleterious (0) | 0,99 | nonsynonymous SNV | 736 | 53 | 37 | 16 | 30 | unknown | unknown | unknown |
| APL_5 | CCDC142 | NM_032779 | 2 | 74702487 | 74702487 | C | T | c.G1640A | p.R547H | 0 | SNV | deleterious (0,15) | 0,45 | nonsynonymous SNV | 0 | 60 | 34 | 26 | 43 | unknown | unknown | unknown |
| APL_5 | CRIP3 | NM_206922 | 6 | 43273860 | 43273860 | A | T | c.T498A | p.H166Q | 0 | SNV | deleterious (0) | 1,00 | nonsynonymous SNV | 407 | 25 | 17 | 8 | 32 | unknown | unknown | unknown |
| APL_5 | DACH2 | NM_053281 | X | 85950147 | 85950147 | T | TGC | c.896_897insGC | p.L299fs | 0 | INS | unknown | unknown | frameshift insertion | 508 | 19 | 14 | 5 | 26 | unknown | unknown | unknown |
| APL_5 | ETNK1 | NM_018638 | 12 | 22826436 | 22826436 | G | A | c.G1054A | p.V352M | 0 | SNV | deleterious (0,07) | 0,02 | nonsynonymous SNV | 517 | 101 | 79 | 22 | 22 | unknown | unknown | unknown |
| APL_5 | GPRIN2 | NM_014696 | 10 | 46999591 | 46999591 | C | CATGAGGGAG | c.711_712insATGAGGGAG | p.G237delinsGMRE | 0 | INS | unknown | unknown | nonframeshift insertion | 0 | 15 | 6 | 9 | 60 | unknown | unknown | unknown |
| APL_5 | RFK | NM_018339 | 9 | 79003515 | 79003515 | C | T | c.G292A | p.A98T | 0 | SNV | deleterious (0,11) | 0,004 | nonsynonymous SNV | 625 | 17 | 10 | 7 | 41 | unknown | unknown | unknown |
| APL_5 | ROCK1 | NM_005406 | 18 | 18586751 | 18586752 | AC | A | c.1547-1G | - | 0 | DEL | 0 | unknown | nonframeshift substitution | 644 | 32 | 25 | 7 | 22 | unknown | unknown | unknown |
