# Supplementary material for: The Mutational Landscape of Acute Promyelocytic Leukemia Reveals an Interacting Network of Co-Occurrences and Recurrent Mutations
Source: PLoS One. 2016 Feb 17;11(2):e0148346. doi: 10.1371/journal.pone.0148346 (PMC4757557; doi:10.1371/journal.pone.0148346)
Supplement: S3 Table — (DOCX) [file pone.0148346.s008.docx]

**Supplementary tables**

**Supplementary table 3**. Main characteristics of the extended cohort of patients.

| **Characteristics** | | n = 25 (100%) |
| --- | --- | --- |
| Male Gender | | 14 (56) |
| Median age (range) | | 11 (44) |
| Leukocyte count x10^9^/L, median (range) | | 12,11 (0,3-59) |
| Hemoglobin g/dL, median (range) | | 9,23 (5,8-12,2) |
| Platelet count x10^9^/L, median (range) | | 36,12 (5-186) |
| Risk group (Sanz et al)^1^ | |  |
|  | Low | 6 (24) |
|  | Intermediate | 12 (48) |
|  | High | 7 (28) |
| Cytogenetics | |  |
|  | t(15;17)(q22;q21) | 14 (56) |
|  | t(15;17)(q22;q21) and additional chromosomal abnormalities | 5 (20) |
|  | cryptic translocation^2^ | 2 (8) |
|  | Non-valuable | 4 (16) |
| *PML-RARA* isoform type | |  |
|  | Bcr-1 | 14 (56) |
|  | Bcr- 2 | 2 (8) |
|  | Bcr- 3 | 7 (28) |
|  | Not available | 2 (8) |
| *FLT3-*ITD mutational status (n=23) | | 4 (17) |
| Treatment Protocol | |  |
|  | LPA-96 | 1 (4) |
|  | LPA-99 | 2 (8) |
|  | LPA-2005 | 19 (76) |
|  | LPA-2012 | 3 (12) |
|  | |  |

^1^ Sanz, M. A., Montesinos, P., Vellenga, E. et al. Risk-adapted treatment of acute promyelocytic leukemia with all-trans retinoic acid and anthracycline monochemotherapy: long-term outcome of the LPA 99 multicenter study by the PETHEMA Group. Blood 2008; 112, 3130-3134.

^2^PML-RARA was diagnosed by FISH or RT-PCR.
