# Supplementary material for: The Mutational Landscape of Acute Promyelocytic Leukemia Reveals an Interacting Network of Co-Occurrences and Recurrent Mutations
Source: PLoS One. 2016 Feb 17;11(2):e0148346. doi: 10.1371/journal.pone.0148346 (PMC4757557; doi:10.1371/journal.pone.0148346)
Supplement: S4 Table — (DOCX) [file pone.0148346.s009.docx]

**Supplementary tables**

**Supplementary table 4.** Genes included in the targeted resequencing.

| **Gene** | **Exons** | **Coverage** |
| --- | --- | --- |
| ABL1 | 14 | 99.14% |
| ADC | 9 | 99.89% |
| AFF2 | 22 | 99.74% |
| AKAP13 | 50 | 99.94% |
| ALPK3 | 14 | 99.97% |
| ARID2 | 24 | 99.89% |
| AURKB | 7 | 100% |
| BAG2 | 5 | 100% |
| BICD1 | 11 | 99.39% |
| C3ORF19 | 11 | 100% |
| CACNA1E | 52 | 99.75% |
| CACNA2D3 | 45 | 100% |
| CELSR1 | 38 | 99.93% |
| CNOT3 | 17 | 99.38% |
| CNTN5 | 28 | 100% |
| COL11A2 | 68 | 99.92% |
| CSMD1 | 82 | 99.7% |
| CSNK1A1L | 1 | 100% |
| CUBN | 70 | 99.94% |
| CUL3 | 23 | 99.82% |
| CYB5D2 | 7 | 98.01% |
| DCT | 11 | 100% |
| DCTN1 | 33 | 99.97% |
| DDR2 | 21 | 100% |
| DIS3 | 22 | 99.91% |
| DNAH9 | 76 | 100% |
| DNPEP | 21 | 99.72% |
| EPHB1 | 29 | 99.76% |
| EPPK1 | 2 | 99.2% |
| ETV6 | 12 | 99.78% |
| EWSR1 | 15 | 99.87% |
| FAM171A1 | 8 | 100% |
| FAM5C | 11 | 99.97% |
| FBLN1 | 26 | 99.63% |
| FILIP1L | 12 | 99.63% |
| FLG | 3 | 86.46% |
| FRG1B | 13 | 50.87% |
| GJB7 | 5 | 98.37% |
| GPR158 | 13 | 100% |
| HDX | 13 | 100% |
| HERC1 | 82 | 99.99% |
| HIVEP1 | 15 | 99.93% |
| HMGCR | 22 | 99.77% |
| IKZF1 | 12 | 100% |
| KIAA0317 | 28 | 99.25% |
| KIDINS220 | 35 | 99.91% |
| KRAS | 7 | 98.24% |
| KRTAP26-1 | 1 | 97.43% |
| LYN | 15 | 100% |
| MAX | 6 | 100% |
| MDN1 | 101 | 100% |
| MIR142 | 1 | 100% |
| MLC1 | 14 | 100% |
| MLL3 | 65 | 97.53% |
| MST1P9 | 17 | 47.92% |
| MYCBP2 | 87 | 99.95% |
| NAV1 | 33 | 98.75% |
| NGLY1 | 13 | 99.63% |
| NOS1 | 36 | 99.01% |
| NR4A2 | 10 | 100% |
| OBSCN | 119 | 99.91% |
| ODZ2 | 37 | 100% |
| OR11H12 | 2 | 59.88% |
| OR5H6 | 1 | 99.14% |
| ORC3 | 23 | 98.22% |
| OVGP1 | 10 | 100% |
| PAPPA2 | 26 | 99.92% |
| PHF6 | 10 | 100% |
| PKD1L2 | 42 | 98.58% |
| PRICKLE2 | 11 | 100% |
| PRPF8 | 42 | 99.7% |
| PRUNE2 | 28 | 99.86% |
| PTPN11 | 19 | 95.79% |
| PTPRG | 32 | 99.89% |
| PTPRT | 34 | 99.95% |
| RBKS | 11 | 99.98% |
| RP1L1 | 6 | 99.38% |
| RUFY1 | 23 | 98.7% |
| RYR3 | 109 | 99.8% |
| SEMG2 | 5 | 100% |
| SHQ1 | 16 | 100% |
| SI | 48 | 99.82% |
| SMC1A | 26 | 100% |
| SMC3 | 30 | 99.69% |
| SRRM2 | 11 | 99.48% |
| STAG2 | 44 | 97.43% |
| TMEM56 | 11 | 98.74% |
| TOP3B | 20 | 93.02% |
| TRIM48 | 6 | 84.99% |
| U2AF1 | 4 | 99.91% |
| UNC5B | 17 | 99.94% |
| USP9X | 46 | 99.84% |
| VCAM1 | 9 | 100% |
| WAC | 16 | 99.96% |
| ZAN | 47 | 97.92% |
| ZNF518B | 4 | 99.62% |
| ZNF788 | 6 | 97.61% |
