# Supplementary material for: The Mutational Landscape of Acute Promyelocytic Leukemia Reveals an Interacting Network of Co-Occurrences and Recurrent Mutations
Source: PLoS One. 2016 Feb 17;11(2):e0148346. doi: 10.1371/journal.pone.0148346 (PMC4757557; doi:10.1371/journal.pone.0148346)
Supplement: S5 Table — (DOCX) [file pone.0148346.s010.docx]

**Supplementary table 5.** The mutated genes detected by target-resequencing.

| **Gene** | **Mutation type** | **Chromosome** | **Start** | **End** | **Nt change** | **AA change** |
| --- | --- | --- | --- | --- | --- | --- |
| AKAP13 | nonsynonymous SNV | 15 | 86213025 | 86213025 | c.A5077C | p.I1693L |
| AKAP13 | nonsynonymous SNV | 15 | 86236554 | 86236554 | c.C71A | p.S24Y |
| AKAP13 | nonsynonymous SNV | 15 | 86122765 | 86122765 | c.C1466A | p.P489Q |
| ALPK3 | nonsynonymous SNV | 15 | 85402489 | 85402489 | c.G4439A | p.R1480Q |
| ALPK3 | nonsynonymous SNV | 15 | 85400223 | 85400223 | c.A2860C | p.N954H |
| ALPK3 | nonsynonymous SNV | 15 | 85360193 | 85360193 | c.C116T | p.P39L |
| ALPK3 | nonsynonymous SNV | 15 | 85383664 | 85383664 | c.G1760T | p.S587I |
| ARID2 | nonsynonymous SNV | 12 | 46245208 | 46245208 | c.T3302G | p.V1101G |
| BICD1 | nonsynonymous SNV | 12 | 32480540 | 32480540 | c.G1151T | p.S384I |
| BICD1 | nonsynonymous SNV | 12 | 32447064 | 32447064 | c.C563T | p.T188M |
| CACNA1E | splicing | 1 | 181726264 | 181726264 |  |  |
| CELSR1 | nonsynonymous SNV | 22 | 46930151 | 46930151 | c.C2917T | p.R973W |
| CELSR1 | nonsynonymous SNV | 22 | 46859960 | 46859960 | c.C3827T | p.P1276L |
| COL11A2 | nonsynonymous SNV | 6 | 33139849 | 33139849 | c.C2699T | p.P900L |
| CSMD1 | nonsynonymous SNV | 8 | 3000194 | 3000194 | c.G6034T | p.A2012S |
| CSNK1A1L | nonsynonymous SNV | 13 | 37679102 | 37679102 | c.G292A | p.E98K |
| CUBN | nonsynonymous SNV | 10 | 16932384 | 16932384 | c.C8741T | p.A2914V |
| CYB5D2 | nonsynonymous SNV | 17 | 4058126 | 4058126 | c.G550T | p.G184C |
| DCTN1 | nonsynonymous SNV | 2 | 74594886 | 74594886 | c.C1719G | p.H573Q |
| DDR2 | nonsynonymous SNV | 1 | 162722945 | 162722945 | c.C143A | p.A48D |
| DNAH9 | nonsynonymous SNV | 17 | 11532747 | 11532747 | c.C1364T | p.T455M |
| EPPK1 | nonsynonymous SNV | 8 | 144941832 | 144941832 | c.G5590T | p.V1864F |
| EPPK1 | nonsynonymous SNV | 8 | 144940580 | 144940580 | c.T6842G | p.L2281R |
| EPPK1 | nonsynonymous SNV | 8 | 144940706 | 144940706 | c.G6716A | p.R2239H |
| EWSR1 | nonsynonymous SNV | 22 | 29693915 | 29693915 | c.G1225A | p.G409S |
| FAM5C | nonsynonymous SNV | 1 | 190234052 | 190234052 | c.C561A | p.D187E |
| FILIP1L | nonsynonymous SNV | 3 | 99568118 | 99568118 | c.A1682C | p.Q561P |
| FILIP1L | nonsynonymous SNV | 3 | 99569387 | 99569387 | c.G413A | p.R138H |
| FILIP1L | nonsynonymous SNV | 3 | 99569382 | 99569382 | c.C418A | p.L140I |
| FLG | nonsynonymous SNV | 1 | 152275345 | 152275345 | c.T12017C | p.V4006A |
| FLG | nonsynonymous SNV | 1 | 152275685 | 152275685 | c.G11677A | p.E3893K |
| FLG | nonsynonymous SNV | 1 | 152286656 | 152286656 | c.T706C | p.Y236H |
| GPR158 | nonsynonymous SNV | 10 | 25861582 | 25861582 | c.T1519G | p.L507V |
| HERC1 | nonsynonymous SNV | 15 | 64040022 | 64040022 | c.A2255C | p.D752A |
| HERC1 | nonsynonymous SNV | 15 | 64066999 | 64066999 | c.C824T | p.S275L |
| HERC1 | nonsynonymous SNV | 15 | 64067311 | 64067311 | c.C512T | p.A171V |
| HMGCR | stopgain SNV | 5 | 74655833 | 74655833 | c.C2322A | p.C774X |
| KIAA0317 | nonsynonymous SNV | 14 | 75137490 | 75137490 | c.G1583A | p.R528Q |
| KRAS | nonsynonymous SNV | 12 | 25398281 | 25398281 | c.G38A | p.G13D |
| MAX | nonsynonymous SNV | 14 | 65560457 | 65560457 | c.G113A | p.R38Q |
| MDN1 | nonsynonymous SNV | 6 | 90418302 | 90418302 | c.T7811G | p.L2604R |
| MDN1 | nonsynonymous SNV | 6 | 90395629 | 90395629 | c.C11636T | p.S3879L |
| MLL3 | nonsynonymous SNV | 7 | 151841826 | 151841826 | c.A14315G | p.N4772S |
| MLL3 | nonsynonymous SNV | 7 | 151902197 | 151902197 | c.G3955C | p.D1319H |
| MST1P9 | ncRNA_exonic | 1 | 17081462 | 17081462 |  |  |
| MST1P9 | ncRNA_exonic | 1 | 17083314 | 17083314 |  |  |
| MST1P9 | ncRNA_exonic | 1 | 17082081 | 17082081 |  |  |
| MYCBP2 | nonsynonymous SNV | 13 | 77900832 | 77900832 | c.A79C | p.T27P |
| MYCBP2 | nonsynonymous SNV | 13 | 77786190 | 77786190 | c.G3163T | p.G1055C |
| MYCBP2 | nonsynonymous SNV | 13 | 77862418 | 77862418 | c.G472A | p.V158I |
| NAV1 | nonsynonymous SNV | 1 | 201778598 | 201778598 | c.G3152T | p.G1051V |
| NAV1 | nonsynonymous SNV | 1 | 201687607 | 201687607 | c.G950A | p.R317H |
| NGLY1 | nonsynonymous SNV | 3 | 25773869 | 25773869 | c.G1312C | p.G438R |
| NR4A2 | stopgain SNV | 2 | 157186322 | 157186322 | c.C377A | p.S126X |
| OBSCN | stopgain SNV | 1 | 228469702 | 228469702 | c.G8266T | p.E2756X |
| OBSCN | nonsynonymous SNV | 1 | 228564890 | 228564890 | c.C23177A | p.P7726Q |
| OBSCN | nonsynonymous SNV | 1 | 228560440 | 228560440 | c.G21961A | p.E7321K |
| OBSCN | nonsynonymous SNV | 1 | 228466473 | 228466473 | c.C6943T | p.R2315W |
| OBSCN | nonsynonymous SNV | 1 | 228482697 | 228482697 | c.C11612T | p.S3871L |
| OBSCN | nonsynonymous SNV | 1 | 228557713 | 228557713 | c.G20038A | p.V6680M |
| ODZ2 | nonsynonymous SNV | 5 | 167631385 | 167631385 | c.C3556A | p.H1186N |
| ODZ2 | nonsynonymous SNV | 5 | 167673910 | 167673910 | c.C5939T | p.P1980L |
| ODZ2 | nonsynonymous SNV | 5 | 167671620 | 167671620 | c.C5689T | p.R1897C |
| PAPPA2 | splicing | 1 | 176664997 | 176664997 |  |  |
| PKD1L2 | nonsynonymous SNV | 16 | 81248710 | 81248710 | c.G553A | p.G185R |
| PRPF8 | nonsynonymous SNV | 17 | 1578592 | 1578592 | c.G2914A | p.E972K |
| PRUNE2 | splicing | 9 | 79319676 | 79319676 |  |  |
| PRUNE2 | nonsynonymous SNV | 9 | 79253144 | 79253144 | c.G8789T | p.S2930I |
| PRUNE2 | nonsynonymous SNV | 9 | 79318472 | 79318472 | c.T8057C | p.L2686S |
| PRUNE2 | nonsynonymous SNV | 9 | 79318262 | 79318262 | c.G8267T | p.R2756I |
| PTPRT | nonsynonymous SNV | 20 | 40733257 | 40733257 | c.C3492A | p.S1164R |
| PTPRT | nonsynonymous SNV | 20 | 40735470 | 40735470 | c.G3346A | p.V1116M |
| PTPRT | nonsynonymous SNV | 20 | 41101155 | 41101155 | c.C1201T | p.R401W |
| PTPRT | nonsynonymous SNV | 20 | 40713356 | 40713356 | c.G4102T | p.D1368Y |
| RYR3 | nonsynonymous SNV | 15 | 33952507 | 33952507 | c.G4505A | p.R1502H |
| SI | nonsynonymous SNV | 3 | 164758807 | 164758807 | c.A2080G | p.T694A |
| SMC1A | nonsynonymous SNV | X | 53436388 | 53436388 | c.G1301A | p.R434Q |
| SRRM2 | nonsynonymous SNV | 16 | 2811610 | 2811610 | c.C1081T | p.P361S |
| STAG2 | nonsynonymous SNV | X | 123195631 | 123195631 | c.T1545A | p.D515E |
| TMEM56 | nonsynonymous SNV | 1 | 95657195 | 95657195 | c.G563A | p.R188Q |
| TRIM48 | nonsynonymous SNV | 11 | 55032782 | 55032782 | c.G451A | p.E151K |
| TTLL5 | nonsynonymous SNV | 14 | 76243133 | 76243133 | c.A2327C | p.E776A |
| U2AF1 | nonsynonymous SNV | 21 | 44515806 | 44515806 | c.G247A | p.E83K |
| UNC5B | nonsynonymous SNV | 10 | 73055656 | 73055656 | c.G2264A | p.R755H |
| USP9X | stopgain SNV | X | 41075269 | 41075269 | c.G5449T | p.E1817X |
| WAC | splicing | 10 | 28878782 | 28878782 |  |  |
| ZAN | nonsynonymous SNV | 7 | 100349931 | 100349931 | c.C2203A | p.P735T |
| ZAN | nonsynonymous SNV | 7 | 100350432 | 100350432 | c.C2704A | p.P902T |
| ZAN | nonsynonymous SNV | 7 | 100331831 | 100331831 | c.G40T | p.A14S |
| ZAN | nonsynonymous SNV | 7 | 100350117 | 100350117 | c.T2389C | p.S797P |
| ZNF518B | nonsynonymous SNV | 4 | 10447523 | 10447523 | c.A430G | p.T144A |
| ZNF788 | ncRNA_exonic | 19 | 12223967 | 12223967 |  |  |
